# Large-scale investigations of Neolithic settlement dynamics in Central Germany based on machine learning analysis: A case study from the Weiße Elster river catchment

**DOI:** 10.1371/journal.pone.0265835

**Published:** 2022-04-20

**Authors:** Jan Johannes Miera, Karsten Schmidt, Hans von Suchodoletz, Mathias Ulrich, Lukas Werther, Christoph Zielhofer, Peter Ettel, Ulrich Veit

**Affiliations:** 1 Institute for Pre- and Early History, University of Leipzig, Leipzig, Germany; 2 Institute for Pre- and Early History, Friedrich-Schiller-University Jena, Jena, Germany; 3 eScience-Center, University of Tübingen, Tübingen, Germany; 4 Institute of Geography, Leipzig University, Leipzig, Germany; 5 Institute for Prehistoric and Medieval Archaeology, University of Tübingen, Tübingen, Germany; Universita degli Studi di Milano, ITALY

## Abstract

The paper investigates potentials and challenges during the interpretation of prehistoric settlement dynamics based on large archaeological datasets. Exemplarily, this is carried out using a database of 1365 Neolithic sites in the Weiße Elster river catchment in Central Germany located between the southernmost part of the Northern German Plain and the Central Uplands. The recorded sites are systematically pre-processed with regard to their chronology, functional interpretation and spatial delineation. The quality of the dataset is reviewed by analyzing site distributions with respect to field surveys and modern land use. The Random Forests machine learning algorithm is used to examine the impact of terrain covariates on the depth of sites and pottery preservation. Neolithic settlement dynamics are studied using Site Exploitation Territories, and site frequencies per century are used to compare the intensity of land use with adjacent landscapes. The results show that the main trends of the Neolithic settlement dynamics can be derived from the dataset. However, Random Forests analyses indicate poor pottery preservation in the Central Uplands and a superimposition of Neolithic sites in the southernmost part of the Northern German Plain. Throughout the Neolithic the margins between soils on loess and the Weiße Elster floodplain were continuously settled, whereas only Early and Late Neolithic land use also extended into the Central Uplands. These settlement patterns are reflected in the results of the Site Exploitation Territories analyses and explained with environmental economic factors. Similar with adjacent landscapes the Middle Neolithic site frequency is lower compared to earlier and later periods.

## 1 Introduction

Humans always depended on natural resources and environmental conditions and altered Holocene landscapes themselves by different activities such as settlement and infrastructure construction, fire ignition, tilling, herding or mining on various time scales [1–4]. Therefore, the diachronic reconstruction of former human activity in a region is crucial to understand former human-environmental interactions. There are two different methodological approaches to reconstruct former human impact in a region: (i) Using geoscientific methods, regional human impact is often derived from sediment archives using paleoenvironmental proxies such as pollen, biomarker, charcoal abundance or gastropods [5–8]. However, although such studies often show a high temporal resolution, upscaling is difficult and they hardly give information about spatial patterns of former human activity. (ii) Spatially resolved information can be obtained by the systematic study of regional settlement patterns based on archaeological data [9–12]. In Germany, a systematic recording of prehistoric sites in inventories, catalogues and maps started at the beginning of the 20th century against the background of an increasing institutionalisation of archaeological research and heritage management [13–15]. This enabled archaeologists to conduct large-scale diachronic studies, i.e. to investigate the prehistoric settlement dynamics in various landscapes by comparing maps with site distributions from different time periods [16, 17]. Most of these studies used archaeological and geographic data to discuss long-term changes in the interactions between early farming societies and their environments. Usually, this was achieved by plotting archaeological sites against different types of soils and land cover [18–28].

In principle, this research approach is still being pursued to the present day. However, the methods applied have been improved: (1) additional terrain covariates are used to investigate settlement patterns. Until today, the most frequently studied terrain covariates include elevation, relief position, slope, exposition, distance to rivers, precipitation, temperature and soil varieties [12, 29]. Occasionally, soil fertility, usable field capacity as well as cation exchange capacity and the suitability of a soil for ploughing are taken into account as well [30–32]. (2) Furthermore, there was a shift towards statistical analyses to improve the reproducibility of research results [33–37]. (3) In addition, standard methods have been established to enable an evaluation of the representativity of archaeological maps. These include a semi-quantitative review of the local research history, a discussion of site distributions with respect to modern land use, the nature and intensity of archaeological field work as well as statistical investigations on the impact of erosion, colluviation and weathering conditions on the preservation of prehistoric sites [38–40]. Furthermore, comparisons of archaeological site distributions to random point distributions as well as (predictive) modelling approaches are applied to the study of former settlement dynamics [11, 37, 41–43]. (4) Finally, there was a shift from spatial analyses based on point coordinates to studies based either on Site-Catchment Analysis or Site Exploitation Territories (SET) [12, 44–51].

**Fig 1:**
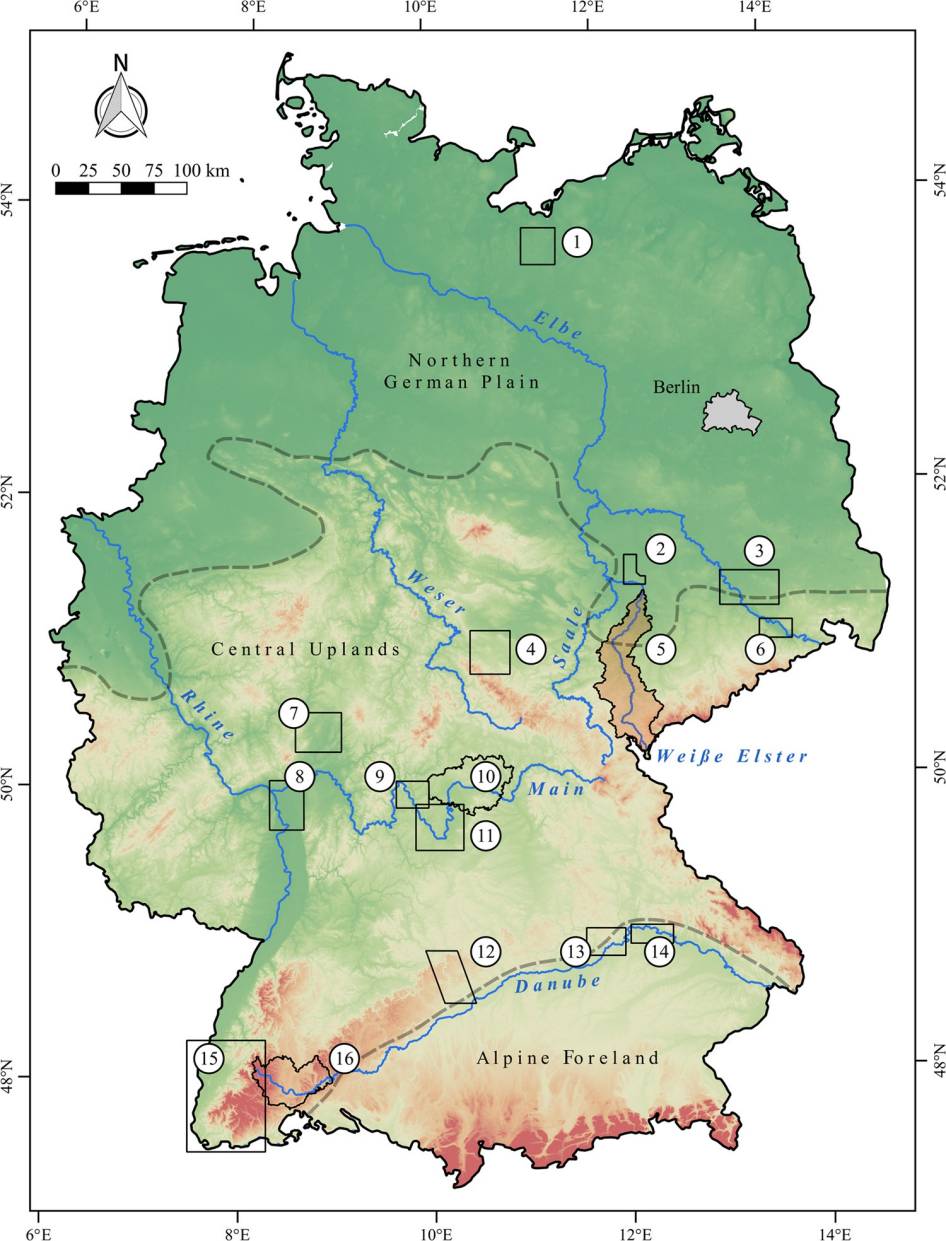
Diachronic studies with a focus on statistical analyses of long-term human-environment interactions between the Neolithic and the middle ages in Germany. (1) Region between Lake Schwerin and Stepenitz [58], (2) north-west Saxony [57], (3) former district of Riesa-Großenhain [56], (4) Gotha landscape [33], (5) Weiße Elster river catchment (present project), (6) widening of the Elbe valley at Dresden [41], (7) northern Wetterau [59], (8) district of Groß-Gerau [29], (9) north-western Main triangle [55], (10) eastern landscape of Lower Franconia [54], (11) southern Main triangle [9], (12) Brenz-Kocher valley in the eastern Swabian Jura [10], (13) lower valley of the Altmühl river [53], (14) Danube valley near Regensburg [52], (15) southern Upper Rhine valley [32] and (16) the Baar and adjacent landscapes [12]. Furthermore, the three main landscape units of Germany are shown. Rivers are derived from the European Catchments and Rivers network system [60]. The topography is based on the SRTM 90 m Digital Elevation Database version 4.1 [61–63].

Up to now, a large number of diachronic studies dealing with long-term human-environment interactions between the Neolithic and the Middle Ages has been carried out (Fig 1). However, these are distributed unevenly across Germany, with a focus on landscapes along the rivers Danube [10, 12, 52, 53] and Main [9, 29, 54, 55] as well as in Central Germany [33, 41, 56, 57]. So far, only one such study has been carried out in the Northern German Plain [58]. Aside from their uneven spatial distribution, it is striking that most studies hardly refer to each other, e. g. regional results are rarely discussed in the context of supra-regional developments [10, 12, 29, 32].

The present study was carried out in the frame of an interdisciplinary geoarchaeological research project that systematically investigates the interplay between the Holocene geomorphological floodplain and slope dynamics, variations of the prehistoric and medieval settlement dynamics and climate changes in large parts of the loess-covered catchment of the Weiße Elster river in Central Germany [64, 65]. We present first results from the large-scale prehistoric to medieval database that was built up during this project, and discuss potentials and challenges during the interpretation of former settlement data exemplarily for the Neolithic. Furthermore, we compare our results with observations from similar studies in adjacent landscapes. Accordingly, the research presented in this paper focuses on the following objectives:

- assessing the current state of knowledge on the Neolithic chronology and culture sequence in the study area by reviewing the local history of (field) research
- evaluating Early, Middle and Late Neolithic site distributions with regard to archaeological field surveys and modern land use
- analysing the interrelation between several terrain covariates, and the depth of Neolithic sites as well as local conditions for the preservation of Neolithic pottery and stone tools, to assess possible influences of geomorphological terrain dynamics and weathering on the archaeological record
- identifying possible biases in the archaeological record
- analysing Early, Middle and Late Neolithic settlement dynamics by using site densities, SETs and site frequencies
- discussing the results in the light of observations from earlier studies in Central Germany

## 2 Study area

**Fig 2:**
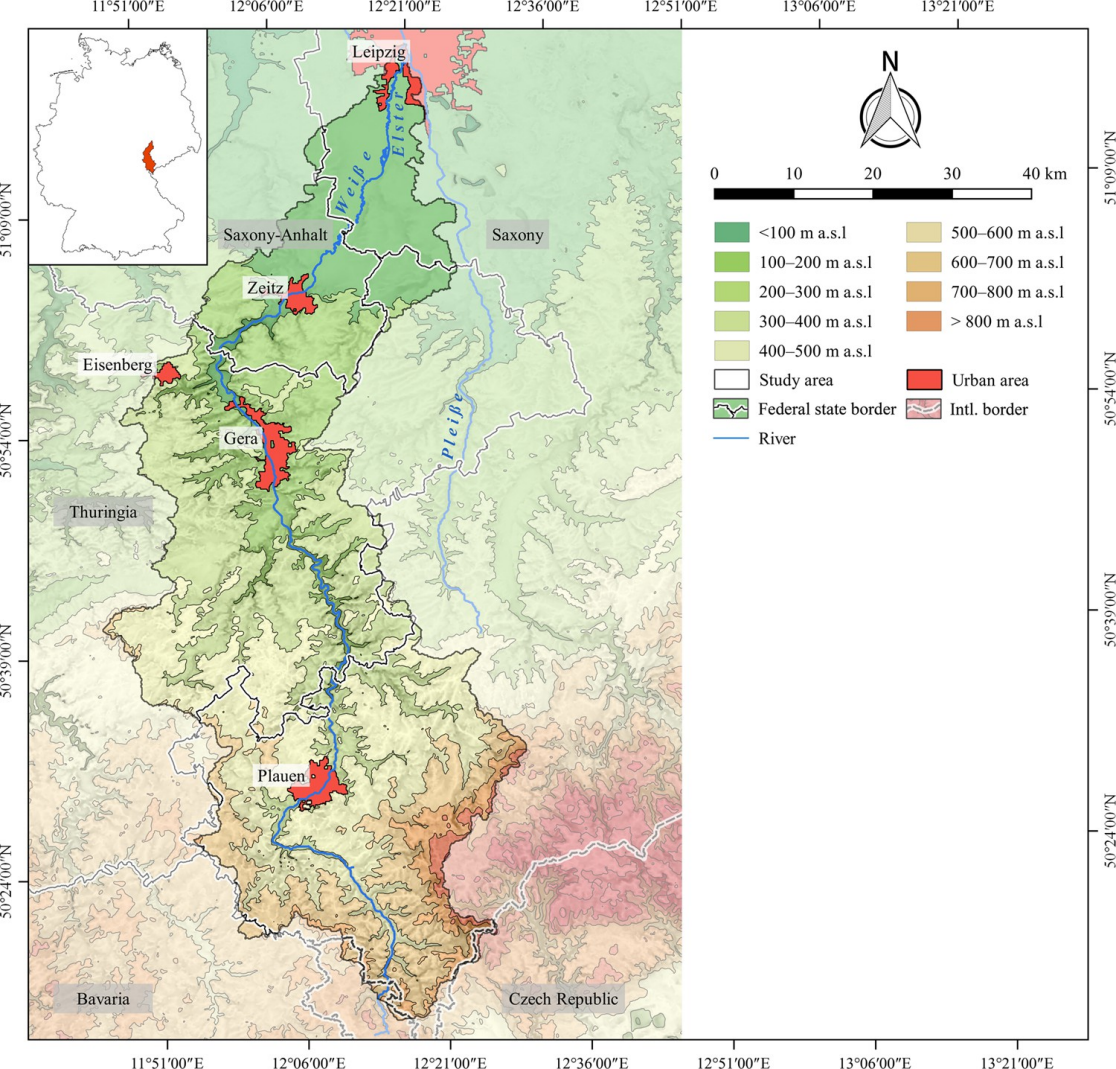
Study area with respect to borders of federal states, international (intl.) borders, local topography and large urban areas mentioned in the text. The outlines of the study area were extracted from the Catchment Characterisation Model database [82]. The political borders are drawn according to the Saxon State Office for Environment, Agriculture and Geology, the Saxony-Anhalt State Office for Surveying and Geoinformation and the Thuringian State Office for Land Management and Geoinformation. Rivers and urban areas are derived from the urban morphological zones dataset and the European Catchments and Rivers network system [83]. The topography is based on the SRTM 90 m Digital Elevation Database version 4.1 [61–63]. Topography and rivers have been modified according to older topographic maps (S2 Fig).

The study area is situated in Central Germany along the borders of the federal states Saxony, Saxony-Anhalt and Thuringia (Fig 2). It is defined by the immediate catchment of the Weiße Elster river between the city of Leipzig in the north, and the German-Czech border in the Elster Mountains (German: Elstergebirge) in the south. It covers an area of approx. 3026 km^2^. Geographically, it is divided into two distinctive landscapes: the northern part belonging to the southernmost part of the Northern German Plain (German: Norddeutsches Tiefland), and the southern part in the Central Uplands (German: Mittelgebirgsschwelle) (S1 Fig) [66–69]. Geologically, the southern part of the Northern German Plain correlates with Cenozoic deposits, whereas Mesozoic to Palaeozoic formations form the southern Central Uplands [70–72]. In the northern part, the average elevation ranges between 100 and 200 m above sea level (a.s.l.), and gently rolling slopes, fertile chernozem/phaeozem and luvisol soils on loess are found [73–75]. The mean annual air temperatures range around 10°C and the mean annual precipitation range between 550 and 650 mm [76, 77]. In the southern Central Uplands, the elevation rises up to 700 m a.s.l., resulting in lower mean annual air temperatures (9–7°C), increasing amounts of annual precipitation (up to 1050 mm) as well as longer periods of winter and frost [76–79]. Here, the topography is more accentuated with deeply cut river valleys and steep slopes. Furthermore, the landscape upstream is less favourable for agricultural use due to low-yielding dystric cambisols and stagnic gleysols towards higher altitudes [80, 81].

## 3 Data and methods

### 3.1 Data

#### 3.1.1 Archaeological dataset and (geo-)statistical tools

To study prehistoric and medieval settlement dynamics in the study area, an archaeological database was built up. We recorded ca. 3100 sites between 2017 and 2020 using archaeological publications and local area files (German: Ortsakten) from the Saxonian Archaeological Heritage Office, the State Office for Preservation of Monuments and Archaeology Saxony-Anhalt, and the Thuringian State Office for Heritage Management and Archaeology. The database covers the time span from the Palaeolithic until 1200 CE, including 1365 sites that can be used to analyse Early, Middle and Late Neolithic settlement dynamics (S1 Dataset). These were investigated using the System for Automated Geoscientific Analyses [84] and QGIS [85].

#### 3.1.2 Pre-processing of archaeological data

**Fig 3:**
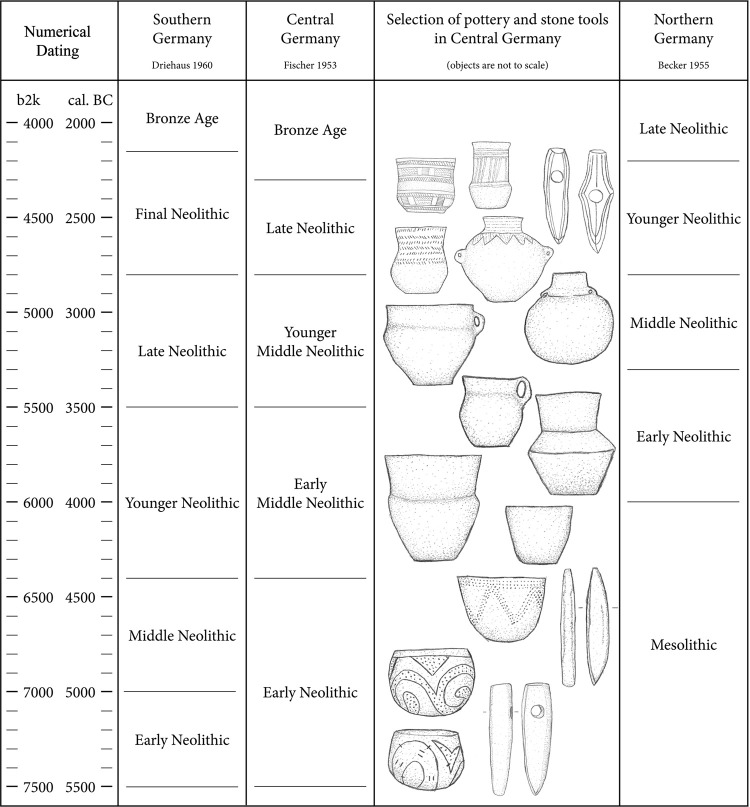
Terminology and chronology of Neolithic periods in Germany. For the Neolithic in Central Germany a selection of characteristic pottery and stone tools is added, drawings by Jan Miera.

The identification of changes in the prehistoric settlement pattern requires archaeological data with a high chronological resolution. Therefore, the dating of each site was registered on different levels: epoch, period, phase and sub-phase [86, 87]. The archaeological sites were classified according to the chronological system for the Neolithic in Central Germany [88–98] (Fig 3).

To analyse the spatial distribution of the recorded sites, we distinguished between sites with an accurate and a symbolic location. The first category applies to sites that can be located in the field. Symbolic coordinates were assigned to sites that cannot exactly be located due to missing or poor geographic information. In these cases, either the presumed location or the church of the village in whose district the site was discovered was taken as the point of reference [99].

Based on the information given in the local area files and literature, three types of sites were differentiated: (I) Settlements are indicated by pits, post-holes, grinding stones, loom weights, spindle whorls or pottery fragments. (II) Burial sites were defined by the presence of human remains. (III) Stray finds included single objects or assemblages with little or no context information. Accordingly, these finds may be either lost objects or material remains from settlements or burial sites that have not yet been identified as such [58, 100]. Therefore, a site was registered twice in our database whenever a burial was documented in a settlement.

Due to frequent construction measures and/or archaeological field work in a relatively small area, the residuals of a settlement or burial site may be discovered in multiple spots close to each other. If each of these archaeological observations were taken as hints to an independent site, the erroneous impression of a densely settled area might emerge, when in fact only the intensity of archaeological observations is reflected. Therefore, we aggregated sites that (I) were less than 250 meters apart from each other, (II) associated with the same or a compatible dating (e.g. “Neolithic” + “Neolithic”, Early Neolithic + “Neolithic”, etc.), and (III) interpreted in the same or a compatible manner (e.g. settlement + settlement, settlement + stray finds, etc.). Since the actual spatial extent of prehistoric sites remains unknown until they have been excavated individually, it is important to acknowledge that this aggregation is ultimately based on an arbitrarily chosen standard. Nonetheless, without this measure analyses of site distribution patterns are prone to result in an overrating of geographical parameters [10, 32, 101, 102].

#### 3.1.3 Pre-processing of spatial data

With respect to the spatial resolution of the archaeological data, continuous raster data and categorical grids derived from vector data were resampled to a resolution of 250×250 m (S1 Table). In addition, corrections of the applied digital elevation model (DEM) [81] were necessary, because the topography south of Leipzig has been altered by modern open-cast mines [103, 104]. Therefore, we georeferenced pre-mining topographic maps (S2 Table) and digitized their contour lines. Thus, we were able to remove remnants from open-cast-mines such as pits, fills or mounds in an area of approx. 640 km^2^ (S2 Fig). In addition, the data for water bodies and rivers were edited based on the georeferenced older topographic maps. This was necessary since several river courses were altered as a result of the open-cast-mines, and because the datasets also contained artificial water bodies that cannot be used for an analysis of the prehistoric settlement dynamics (S3 Fig). The result is an approximation to prehistoric and medieval conditions, as the exact course of each river is subject to continuous changes [105, 106].

#### 3.1.4 Archaeological source criticism

To evaluate Neolithic site distribution patterns and thus the reliability of the results that can be derived from them, an analysis of the archaeological data is necessary. In principle, it must be assumed that the distribution of prehistoric sites is influenced by (I) archaeological filters, i.e. the type and intensity of archaeological field work, (II) geographical filters such as modern land use, erosion, colluviation or weathering and (III) culturally intrinsic filters, i.e. the actual understanding of land use strategies in prehistory. The aim of archaeological source criticism is therefore to approximate the degree of distortion caused by the first two filters, and thus to evaluate the overall quality of the archaeological database [86, 107–109].

### 3.2. Archaeological filters

#### 3.2.1 State of local research

The local state of archaeological research will be presented in the form of key data describing the basic composition of the Neolithic record according to our database. These include the number of recorded sites with respect to their dating, the interpretation of their individual function, and the accuracy of their location as well as their status of publication [9–12, 32, 53–59].

#### 3.2.2 Intentionality of site discoveries

It is possible to distinguish between intentional and non-intentional modes of site discoveries: The first category includes archaeologically motivated activities such as excavations, field surveys or aerial photography. In the second category, activities without archaeological motivation are subsumed, including construction measures, the extraction of raw materials (mining) or activities related to agriculture and forestry [110]. The discrimination between these categories is important, because non-intentional modes of discovery represent a random sample with regard to the spatial and chronological distribution of prehistoric sites. Accordingly, the analysis of the intentionality is crucial for the understanding of the representativeness of the dataset [9].

Furthermore, territories of individual collectors might affect site distribution patterns. Therefore, it is necessary to discuss the Neolithic site distribution with respect to areas that were investigated by field surveys or aerial photography [10, 12, 32, 39]. Taking into account individuals who discovered more than nine sites [100], we modelled the territories covered by each method separately from each other on the basis of the largest empty circle [111–113]. This discrimination was done because aerial photography may result in the discovery of archaeological features on or slightly below the recent surface. However, in contrast to field surveys, this method cannot contribute to the discovery of small stone tools and pottery fragments. For each approach, the investigated territories were described by 1.5 and 2.5 km isolines. It should be borne in mind that the modelled territories were derived from data of surveys that successfully resulted in site discoveries. Accordingly, the modelled territories are not necessarily congruent with the areas actually surveyed, but rather smaller.

### 3.3 Geographical filters

#### 3.3.1 Site distribution in relation to archaeological site visibility and preservation of material remains

Local geomorphic terrain dynamics are known to affect archaeological site visibility and the preservation of material remains: Firstly, colluviation and alluviation can lead to the superimposition and thus the protection of prehistoric sites at the cost of their visibility [38, 114, 115]. This can be discussed by using information about the level of an archaeological site below the recent surface that can be drawn from the activity leading to its discovery [9, 39, 58, 59]. Secondly, erosion may lead to the uncovering of prehistoric sites, resulting in an improved accessibility at the cost of reduced preservation conditions or even their complete destruction due to an increased exposure to weathering and ploughing [107, 108]. To examine this aspect, we differentiated between sites where pottery (and stone tools) were recorded, and those where only stone tools were discovered.

To discuss possible effects of terrain covariates (S1 Table) on the depth of Neolithic sites as well as the presence/absence of pottery, we used the Random Forests (RF) modelling algorithm, i.e. an ensemble learning method for classification and regression [116]. RF is suited for spatial archaeological research and predictive modelling, because it can handle complex spatial and contextual datasets with weak, imbalanced or noisy inputs. RF consists of a large number of individual decision trees that form an ensemble, where each individual tree is based on a distinct set of binary decision rules. In addition, RF reduces over-fitting by averaging (regression) or aggregating by majority vote (classification) of all individual tree results [117, 118]. A description of the RF algorithm is provided below:

- Initially, a random subset of the original dataset was generated by using the bootstrap method (random sampling with replacement) for each individual tree. Thus, we did not use the entire dataset for learning, but the same number of samples. Usually, the original dataset is represented in the sample set with ~63% of unique and ~37% of redundant samples. Thus, the remaining samples not represented in the training set are called out-of-bag (OOB) samples and used for validation.
- A second-level random component was used to build the individual tree. Therefore, at each node the best binary split on the training data was performed, based on a random subset of predictors (S1 Table). The number of covariates (mtree) can be chosen by the user. However, Breiman [116] suggests to use the square root of the number of predictors. Based on a binary recursive partitioning method a distinct set of binary rules is generated within all individual trees, with the aim to separate each single class (classification case) in a terminal node. This is done until no further partitioning is possible, e.g. a minimum set of samples is represented in a node or the node contains only samples with one single class label (perfect split).
- If used for a classification, the results from each decision tree were aggregated on the basis of a majority vote.

**Fig 4:**
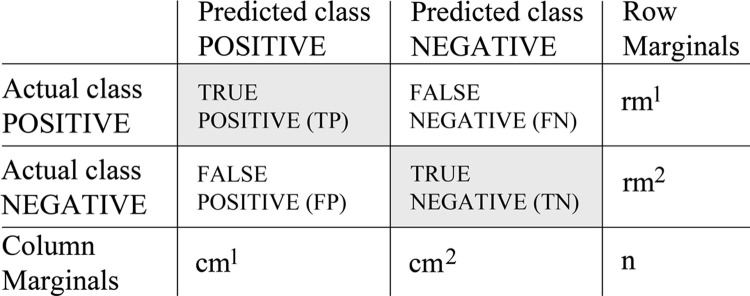
Random forests confusion matrix according to van Rijsbergen [120].

The algorithm provides a distinct set of accuracy measurements to evaluate the performance of the RF model [119]. These are based on the confusion matrix (Fig 4) [120], which consists of four specific conditions (true positive, false positive, false negative, true negative) related to the predicted OOB output. This allows a detailed analysis of the classification accuracy. To avoid misinterpretation, we used different quality indices to evaluate model accuracy and predictive power: OOB error rate [Formula 1], Cohen’s Kappa score [Formula 2], Recall [Formula 3], Precision [Formula 4] and F_1_-measure [Formula 5].

The out-of-bag error rate is calculated as follows:

$$\mathrm{OOB}\;\mathrm{error}\;\mathrm{rate}=\frac{\mathrm{sum}\;\mathrm{of}\;\mathrm{mis}‐\mathrm{classified}\;\mathrm{observations}}{\mathrm{sum}\;\mathrm{of}\;\mathrm{observations}\;\mathrm{in}\;\mathrm{training}\;\mathrm{set}}$$ (1)

The Kappa value measures the agreement between predictions and observed cases. This is achieved by comparing the overall accuracy to an expected random chance accuracy. Therefore, the Kappa score is particularly useful on classifications with imbalanced data [121–124]. The results range from -1 to 1 with the latter being a perfect classification, followed by strong agreement (>0.8), substantial agreement (between 0.4 and 0.8) and poor agreement (<0.4). A score of 0 indicates that the classification is as good as random. Values below 0 point to no effective agreement or even disagreement [125, 126].

Cohen’s Kappa is calculated using the following formula [124]:

$$\kappa =\frac{\mathrm{Pr}(a)-\mathrm{Pr}(e)}{1-\mathrm{Pr}(e)}$$ (2)

Where Pr(a) represents the actual observed agreement (true positives) and Pr(e) chance agreement.


$$\mathrm{Pr}(e)=\frac{\left(\frac{{\mathrm{cm}}^{1}\times {\mathrm{rm}}^{1}}{n})+(\frac{{\mathrm{cm}}^{2}\times {\mathrm{rm}}^{2}}{n}\right)}{n}$$

Where cm^1^ represents column 1 marginal, cm^2^ column 2 marginal, rm^1^ row 1 marginal, rm^2^ row 2 marginal and n the number of observations.

In addition to the described measures of agreement in a categorical use-case (classification), we used additional validation measures by deriving so-called measures of effectiveness such as recall, precision and the F_1_-measure based on the confusion matrix, which are important measures for information retrieval [120, 127].

Recall (Rc) describes the relation between positive (true positive: TP) and negative (false negative: FN) predicted class values, and thus the probability that a pre-classified sample is actually predicted, i.e. it represents underestimation. In a prediction without erroneous assignments (FN), the value is 1. The recall is calculated as follows:

$$\mathrm{Rc}=\frac{\mathrm{TP}}{\mathrm{TP}+\mathrm{FN}}$$ (3)

In contrast, precision (Pc) describes the probability that an estimated prediction result is actually also pre-classified within the training dataset (true negative: TN), i.e. it represents overestimation. In a prediction without erroneous assignments (FP), the value is 1. The precision is calculated as follows:

$$\mathrm{Pc}=\frac{\mathrm{TP}}{\mathrm{TP}+\mathrm{FP}}$$ (4)

To quantify the overall model quality in a composite way the F_1_-measure is frequently used. The F_1_-measure represents the harmonic mean between under- and overestimation, and is calculated as follows:

$$F_{1}=\frac{2*\mathrm{Pc}*\mathrm{Rc}}{\mathrm{Pc}+\mathrm{Rc}}$$ (5)

Additionally, RF provides a measure of importance of each covariate by calculating their mean decrease accuracy [Formula 6], i.e. the impact of each variable on the prediction error when it is not taken into account [127, 128]. This enables in-depth analysis of interaction from the covariate space related to our classification problem. Breiman [116] suggests the difference in prediction accuracy before and after permuting a single covariate, averaged over all trees, as a measure for variable importance. The mean decrease of accuracy is calculated as follows [128]:

$${\mathrm{VI}}^{\left(t\right)}(X_{j})=\frac{\sum I(y_{i}{=y}_{i}^{\left(t\right)})}{\left|{\bar{B}}^{\left(t\right)}\right|}-\frac{\sum I(y_{i}{=y}_{{i,\pi }_{j}}^{\left(t\right)})}{\left|{\bar{B}}^{\left(t\right)}\right|}$$ (6)

where ${\bar{B}}^{\left(t\right)}$ is the out-of-bag sample for a tree, $y_{i}^{\left(t\right)}{=f}^{\left(t\right)}(x_{i})$ the predicted class for observation *i* before, and $y_{{i,\pi }_{j}}^{\left(t\right)}{=f}^{\left(t\right)}(x_{{i,\pi }_{j}})$ the predicted class for observation *i* after permuting its value of variable *X j*, i.e. with $x_{{i,\pi }_{j}}=(x_{i,1},\ldots {,x}_{i,j-1,}x_{\pi_{j}(i),j}{,x}_{i,j+1},\ldots {,x}_{i,p}).$


$$\mathrm{VI}(X_{j})=\frac{\sum {\mathrm{VI}}^{\left(t\right)}(x_{j})}{\mathrm{ntree}}$$

Following Breiman [116], we used the square root of the number of predictors (mtree) and a maximum number of individual trees of 1500. Even though the general precondition of a RF algorithm is robust against multi-collinearity [116, 129, 130], we only used covariates with correlation of less than 0.8 (S1 Table; S4 Fig). To avoid statistical coincidence, all analyses were repeated ten times to calculate average values for the OOB error rate, Kappa, Precision, Recall and F_1_-measures. The statistical analyses were carried out using the packages *randomForest* [131] and *caret* [132, 133] in R version 3.6.3 [134].

#### 3.3.2 Site distribution in relation to modern land use

Various large-scale studies showed that modern land use has an impact on the accessibility and preservation of prehistoric sites [10–12, 32, 39, 42, 135–137]. In recent years, CORINE Land Cover (CLC) raster data have been successfully used to evaluate the relationship between archaeological site distribution patterns and modern land use [10–12, 42]. Based on satellite imagery, the dataset originally discerns 44 classes of modern land use with a spatial r esolution of 100×100 meters [83]. However, it is recommended to aggregate these classes for archaeological purposes [10, 12, 100]. Here, we distinguished seven types of land use: urban areas, forests, arable land, grassland, water bodies, bogs or swamps, and landfills or dumpsites.

We applied the Chi-squared test to assess the interrelation between the relative frequency of land use classes and Neolithic sites. This was achieved by calculating the number of sites expected to be affiliated with each land use class, and then comparing these values with the observed distribution of Neolithic sites over these classes. The significance of the deviations between the expected and observed sites was described by the χ^2^ value, calculated as follows:

$$x^{2}=\sum_{i=1}^{k}\frac{{\left(O_{i}-E_{i}\right)}^{2}}{E_{i}}$$

Where *k* is the number of categories, *O*_i_ the observed number of cases in category *i*, and *E*_i_ the expected number of cases in category *i*. The sum of all χ^2^ values was compared with the critical χ^2^ value p [138–140].

### 3.4 Neolithic settlement dynamics

#### 3.4.1 Site frequency

Due to the fact that the duration of each Neolithic period varies, we used the site frequency per century (German: Fundstellenfrequenz) to enable a comparison of the settlement intensity between the Neolithic periods. It was calculated as follows [34, 48]:

$$\mathrm{Frequency}\;\mathrm{per}\;\mathrm{century}=\frac{S_{n}}{P_{n}}*100$$

Where *S*_*n*_ is the number of sites and *P*_*n*_ is the duration of the respective period in years.

The site frequency can be used to compare varying settlement intensities on a supra-regional scale [12, 39, 141]. However, the comparability between individual study areas is limited due to their varying size, which in turn affects the number of sites that can be recorded. To improve the comparability of study areas of different sizes, we calculated the average site frequency for an area of 250 km^2^. The site frequency was processed as follows:

$$\mathrm{Average}\;\mathrm{site}\;\mathrm{frequency}\;\mathrm{per}\;250\;{\mathrm{km}}^{2}=\frac{\mathrm{Fr}}{\left(\frac{\mathrm{Area}}{250}\right)}$$

Where *Fr* is the local site frequency and *Area* the spatial extent of the respective study area in km^2^.

#### 3.4.2 Site distribution and density

To describe the spatial distribution and density of the recorded Neolithic sites, we calculated 1.5 and 2.5 km isolines based on the largest empty circle between sites with accurate coordinates [111–113]. This approach allowed the identification of core areas of Neolithic settlement on an empirical basis for each period. By comparing the extent and distribution of these areas, the settlement dynamics can be derived [142, 143].

#### 3.4.3 Site Exploitation Territories (SET) and settlement dynamics

We applied the concept of SET to investigate the Neolithic settlement dynamics. This concept was developed to study archaeological sites in the context of their environment [144–146]. The term refers to a time-distance based territory that is presumably visited for daily subsistence [147–149]. Accordingly, the local topography (slope) influences the shape of SETs. In landscapes with gently rolling slopes SETs tend to circular shapes, whereas due irregular forms are more common in mountainous regions [113, 150, 151].

SETs are based on the surmise that each site has an optimal geographic location with respect to its economic function. Consequently, it is expected that mobile groups whose subsistence was pasture farming preferred locations favourable for grazing, whereas settlements from sedentary societies are expected to be located in areas suitable for agriculture [147, 149, 152]. With respect to anthropological field studies, it is assumed that the intensity of resource and land exploitation decreases with increasing distance from the settlement. In the case of sedentary societies, the territory used on a daily subsistence basis is assumed to be in the area that can be reached within one hour’s walking distance [147–149, 152]. However, the most important activities related to arable farming are considered to take place in the immediate vicinity of a settlement, i.e. in an area that can be reached within 10 to 15 minutes walking [148, 149, 153–156]. In our study area this was confirmed by investigations of Neolithic wells from Eythra and Droßdorf. Due to good preservation of organic material, remains of cultivated plants, field weeds and insects were discovered in the wells that indicate agricultural activities in the direct vicinity of the settlements [157–159].

We modelled for all Neolithic sites with accurate coordinates their individual SET that can be reached within ten minutes [160]. The script implements the hiking function developed by Waldo Tobler [161] which has been used before in archaeological studies [162, 163]. This function is an empirical model based on marching data of the Swiss military, taking into account multiple factors such as vegetation, individual physical fitness, length and quality of the path, altitudinal difference, weather conditions, darkness as well as marching competence and luggage [164].

Here, SET modelling was based on processed SRTM DEM data with a resolution of 250×250 meters, an estimated walking distance of 5 km/h on flat terrain as suggested by Tobler [161], and a damping of walking speed on slopes with an inclination of >15° [12]. The settlement dynamics were investigated by comparing two sets of grid statistics for each period: one derived from all sites dating to the respective period, and the other derived exclusively from settlements.

To further characterize the SETs, the following terrain covariates were used: area covered by the SET, elevation above sea level, slope and valley depth (i.e. height above nearest river) as derived from the processed DEM, river distance derived from the processed data and percentage of soils on loess (S1 Table). All these terrain covariates are subject to both natural and anthropogenic changes [105, 106, 165–167]. In an ideal scenario, GIS analyses of prehistoric site distributions are based on individual datasets that are representative for each period. However, as there are no datasets that meet this standard, archaeological studies have to rely on available data [10, 12, 32]. With regard to the preceding remarks, the results of the SET analyses are to be understood as results of model calculations. They represent an approximation to archaeological data, and provide a basis for the discussion of settlement dynamics.

## 4 Results

### 4.1 Archaeological filters

#### 4.1.1 State of local research

Following the systematic processing of all available information from the literature and the local area files, our record of the Neolithic in the study area consists of 1365 sites, including 361 Early, 82 Middle and 298 Late Neolithic sites (S3 Table). Due to the rather unspecific nature of the retrieved artifacts, the remaining 624 sites can only be described as “Neolithic”. The Early Neolithic is represented by 208 settlements, 8 burial sites and 145 stray finds. In contrast, 47 settlements, 19 burial sites and 16 stray finds are associated with the Middle Neolithic. There are 74 settlements, 96 burial sites and 128 stray finds dating to the Late Neolithic. In total, 769 Neolithic sites are exactly located, including 340 settlements, 123 burial sites and 306 stray finds. Symbolic coordinates were assigned to 70 settlements, 26 burial sites and 500 stray finds (S3 Table). Not more than 703 sites have been published until today, which underlines the paramount value of using local area files in large-scale studies (S4 Table).

#### 4.1.2 Intentionality of site discoveries

The mode of discovery is documented for 843 sites (S5 Table). Their majority is associated with non-intentional modes of discovery (n = 527). Construction measures (n = 201) and the extraction of raw materials (n = 182) take the largest share of this group. In contrast, 313 sites are linked to intentional modes of discovery, mainly field surveys (n = 288).

**Fig 5:**
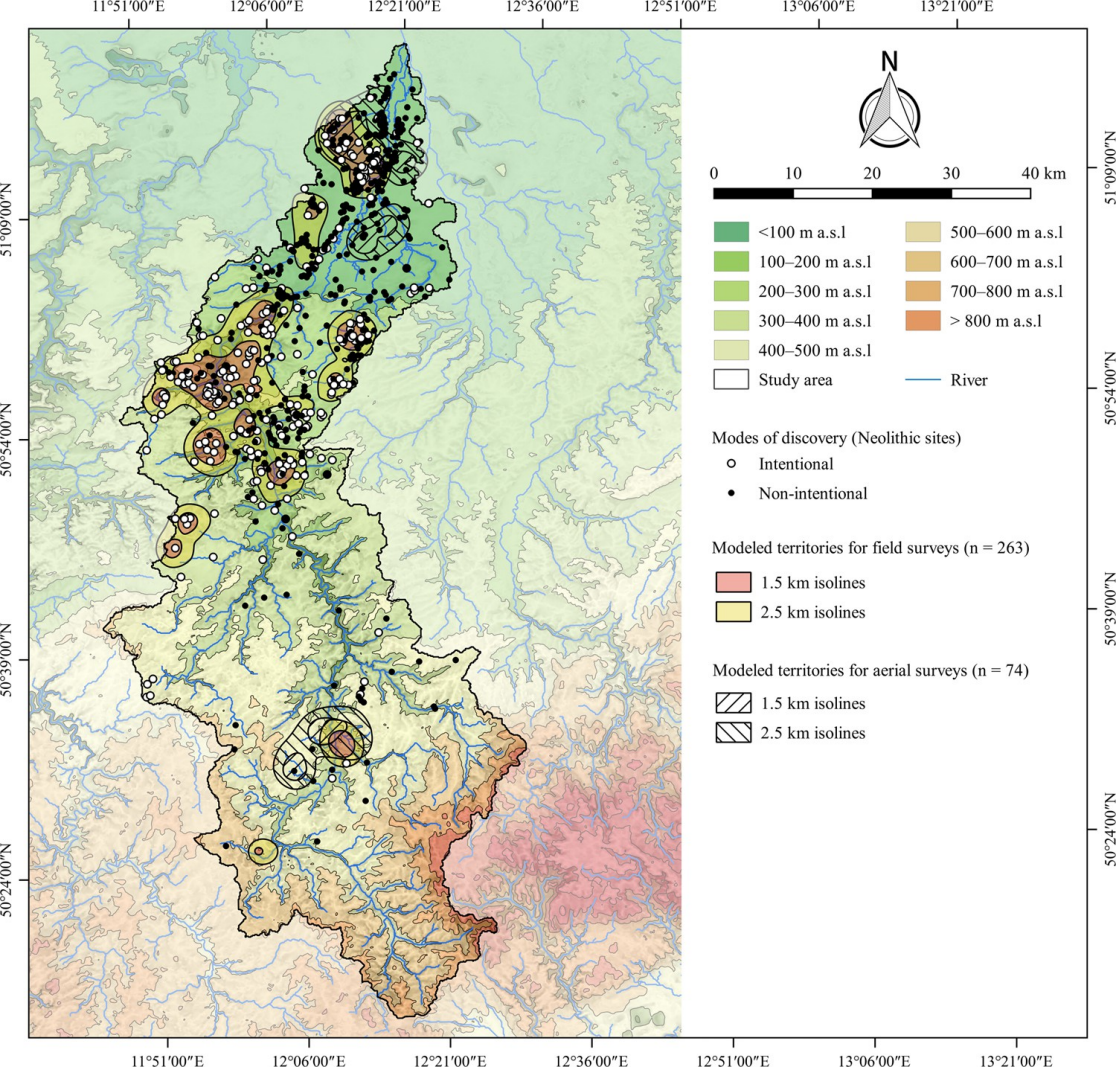
Distribution of Neolithic sites with respect to their modes of discovery against the background of areas investigated by field surveys and aerial photography. Rivers were plotted according to the European Catchments and Rivers network system [60]. The topography is based on the SRTM 90 m Digital Elevation Database version 4.1 [61–63]. Topography and rivers have been modified according to older topographic maps (S2 Table). See Fig 2 for a location of the urban areas mentioned in the text.

Based on the archaeological database, 12 individuals were identified who repeatedly carried out field surveys in the study area and discovered at least nine sites. In total, these individuals registered 263 prehistoric and medieval sites that were used to model the surveyed territories (Fig 5). In total, 125 Neolithic sites were used for modelling the surveyed territories. This represents 30% of all Neolithic sites registered in those territories (n = 422), and 69% of all Neolithic sites discovered on the surface in these areas (n = 182). Accordingly, the field surveys resulted in an increase in the density of Neolithic sites, especially between Gera and Zeitz (Fig 5).

Finally, territories for aerial surveys were calculated on the basis of 74 sites south of Leipzig and in the vicinity of Plauen. However, the aerial surveys did not affect Neolithic site distributions (Fig 5).

### 4.2 Geographical filters

#### 4.2.1 Site distribution in relation to archaeological site visibility and preservation of material remains

In total, there are 302 sites with pottery and 213 sites where only stone tools have been recorded, respectively 251 sites discovered on the surface and 264 sites that were buried at the time of their discovery (Table 1).

**Table 1:** Dataset used in random forests analyses.

Depth of sites	Pottery and stone tools	Stone tools only	Sum
Discovered above the surface	96	155	251
Discovered below the surface	206	58	264
Sum	302	213	515

**Fig 6:**
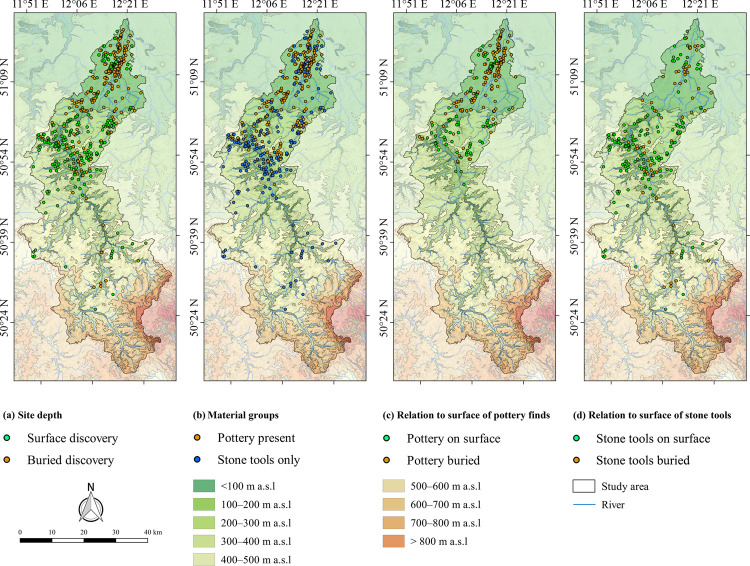
Mapping of datasets used in random forests analyses. (a) Site depth, (b) material groups (c) depth of pottery and (d) stone tools (Table 1). Rivers are plotted according to the European Catchments and Rivers network system [60]. The topography is based on the SRTM 90 m Digital Elevation Database version 4.1 [61–63]. Topography and rivers have been modified according to older topographic maps (S2 Table). See Fig 2 for a location of the urban areas mentioned in the text.

The majority of buried sites is located in the northern study area in the southernmost part of the Northern German Plain, while sites on the surface are more frequent in the Central Uplands (Fig 6A). A comparison of the material groups shows a concentration of sites with pottery in the northern study area. In contrast, in the area around Gera and further south in the Central Uplands there are almost exclusively sites without pottery (Fig 6B). The vast majority of discovered Neolithic pottery in the northern study area was buried at the time of its discovery. In contrast, in the southern Central Uplands Neolithic pottery tended to be on the surface (Fig 6C). The distribution of sites with stone tools but without pottery shows a concentration in the vicinity of Gera, and extends further south into the Central Uplands. A contrast between buried sites and discoveries on the surface is not recognisable (Fig 6D).

The RF model performed well with respect to the depth of the sites, showing an average OOB estimate of the error rate of 21.35%, Kappa score of 0.59, Precision of 0.80, Recall of 0.79 and F_1_ of 0.79 (S6 Table). Therefore, based on the selected terrain covariates (S1 Table) the RF algorithm could reproduce the distribution pattern of the depth of sites (Fig 6A). According to the mean decrease accuracy, the five most important terrain covariates are (1) elevation, (2) copper distribution, (3) the proportion of coarse fragments and (4) sand in the soils as well as (5) the slope length and steepness factor (LS-factor).

In addition, the RF algorithm was able to reproduce the presence-absence-pattern of Neolithic pottery (Fig 6B), and resulted in an average OOB estimate of the error rate of 22.64%, Kappa score of 0.53, Precision of 0.79, Recall of 0.81 and F_1_ of 0.79 (S7 Table). The five most important terrain covariates were (1) elevation, (2) copper distribution, (3) LS-factor, (4) slope and (5) the proportion of coarse fragments in soils.

Furthermore, the RF algorithm was able to reproduce the distributions of pottery with regard to site depth, and resulted in an average OOB estimate of the error rate of 19.40%, Kappa score of 0.55, Precision of 0.75, Recall of 0.63 and F_1_ of 0.68 (S8 Table). Hence, based on the selected terrain covariates the RF algorithm reproduced the classification of buried and non-buried pottery (Fig 6C). The five most important terrain covariates were (1) copper distribution, (2) the proportion of coarse fragments in soils, (3) elevation, the (4) wind erodible fraction of the soil and (5) slope.

With regard to the distribution of (non-)buried stone tools, the RF model resulted in an average OOB estimate of the error rate of 24.54%, Kappa score of 0.21, Precision of 0.75, Recall of 0.89 and F_1_ of 0.81 (S9 Table). Accordingly, the low Kappa score demonstrates that unlike the preceding analyses the RF algorithm could not reproduce the classification of (non-)buried stone tools (Fig 6D) based on the selected terrain covariates.

#### 4.2.2 Site distribution in relation to modern land use

**Fig 7:**
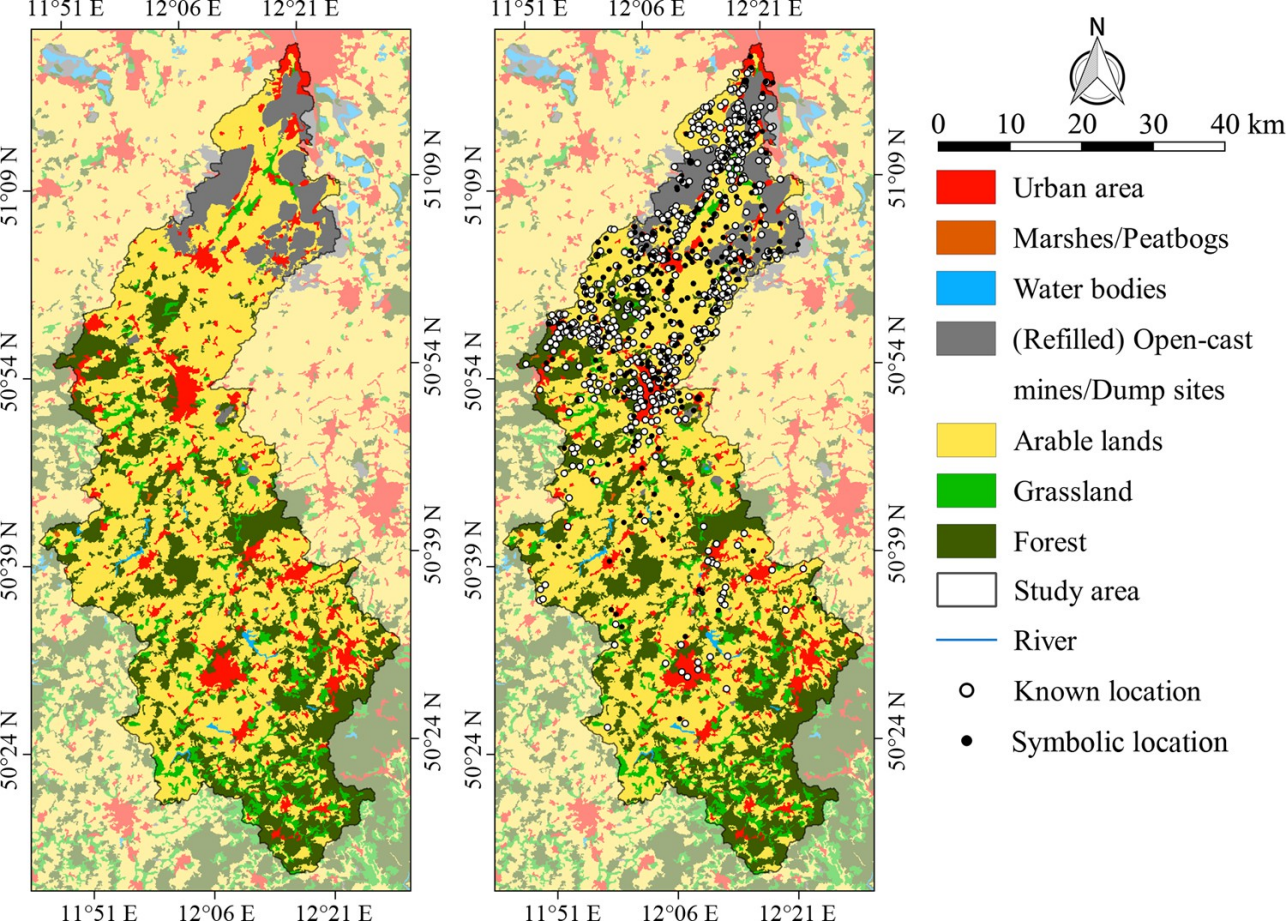
Modern land use and Neolithic site distribution in the study area. Distribution of modern land use classes according to CORINE Land Cover data [83]. The spatial extent of the (refilled) open-cast mines has been modified according to older topographic maps (S2 Table; S2 Fig).

The recorded Neolithic sites are unevenly distributed over modern land use classes (Fig 7). According to the Chi-squared test this observation is statistically significant (Table 2). This is mainly due to the fact that approx. twice as many sites as expected were discovered in urban areas and mining areas. Also on arable land the number of recorded sites exceeds the number of expected sites. In contrast, some land use classes seem to reduce the possibility of discovering Neolithic sites, and thereby also contribute to the uneven site distribution. This is especially true for forest and grassland (Table 2).

**Table 2:** Distribution of Neolithic sites over modern land use.

CORINE Land Cover	Spatial abundance (%)	Recorded sites (n)	Expected sites (n)	X^2^ value	Rec./Exp.
Urban area	9.3	134	72	54.40	1.87
Mining area/Dumpsite	7.3	118	56	68.17	2.10
Arable land	53.1	434	408	1.61	1.06
Grassland	6.0	29	46	6.37	0.63
Forest	23.9	54	184	91.66	0.29
Bog	0.0	0	0	0.00	0.00
Water body	0.4	0	3	3.08	0.00
Sum	100.0	769	769	225.28	

The critical X^2^ value for 6 degrees of freedom is at 22.46 (significance level: 0.001%) [138].

### 4.3 Neolithic settlement dynamics

#### 4.3.1 Site frequency

On average, there is a frequency of approx. 43 sites per century for the entire Neolithic. However, strong fluctuations can be observed for the individual periods. The Early Neolithic is characterized by a frequency of ca. 33 sites per 100 years. The transition to the Middle Neolithic is marked by a decline to 5, followed by a massive increase to ca. 60 with the onset of the Late Neolithic.

#### 4.3.2 Site distribution and density

**Fig 8:**
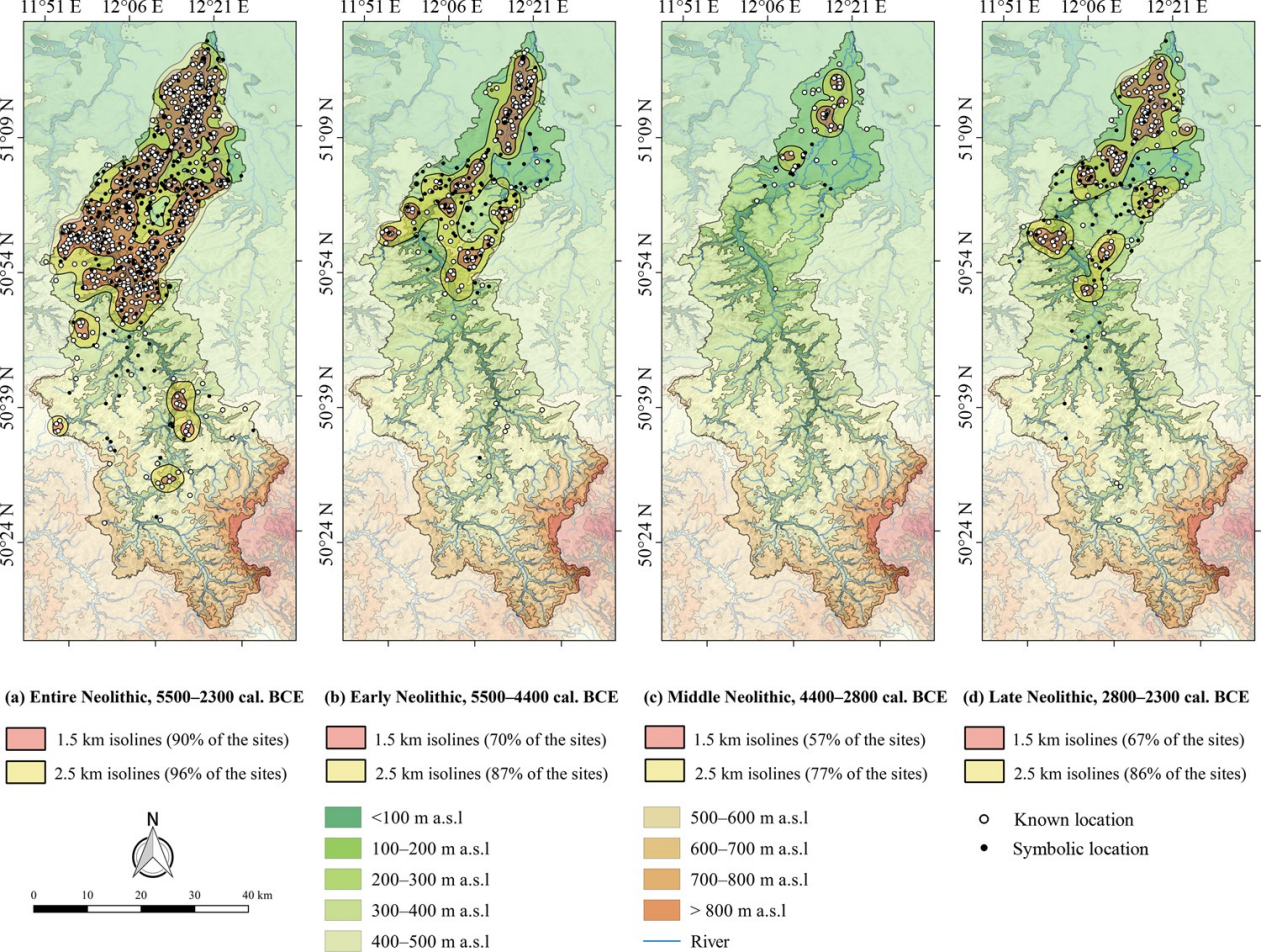
Neolithic site densities in the study area. (a) Entire Neolithic, (b) Early Neolithic, (c) Middle Neolithic and (d) Late Neolithic. Rivers were plotted according to the European Catchments and Rivers network system [60]. The topography is based on the SRTM 90 m Digital Elevation Database version 4.1 [61–63]. Topography and rivers have been modified according to older topographic maps (S2 Table). See Fig 2 for a location of the urban areas mentioned in the text.

The 1.5 km isolines cover about 90% of all Neolithic sites with exact coordinates. Site concentrations are recognisable in the southernmost part of the Northern German Plain south of Leipzig, along the Weiße Elster river between Zeitz and Gera, and north-east of Gera. Furthermore, the 1.5 km isolines describe areas with a locally increased density of sites in the Central Uplands. These are located in the valley of the Weiße Elster near Plauen, north-east of that town, as well as in the area of the watershed to the river Saale. In addition, there is a loose scattering of sites in the Central Uplands which are located outside the 1.5 or 2.5 km isolines (Fig 8A).

Based on the 1.5 km isolines, the distribution of about 70% of the Early Neolithic sites can be described. An elongated concentration south of Leipzig along the Weiße Elster river valley is recognisable. Further site concentrations can be described with the 1.5 km isolines near Zeitz, Eisenberg and north-east of Gera. These local concentrations are connected by the 2.5 km isoline which covers 87% of the Early Neolithic sites. Far outside these isolines there are isolated sites in the Central Uplands (Fig 8B). The majority of the Middle Neolithic sites is loosely scattered, so that the 1.5 km isoline can only cover about 57% of the sites and the 2.5 km isoline about 77%. A rather elongated concentration along the Weiße Elster valley and a small concentration near Zeitz are described. In addition, there are several isolated sites in a greater distance from the Weiße Elster valley. South of Zeitz there are only very few sites dating to the Middle Neolithic (Fig 8C). In comparison, the distribution of Late Neolithic sites covers a larger area than the Middle Neolithic. The 1.5 km isolines cover 67% of the Late Neolithic sites and describe several areas with a higher density: a large area along the Weiße Elster valley and west of it, smaller areas near Zeitz, Eisenberg, Gera, and one on the watershed between the rivers Weiße Elster and Pleiße. Overall, the distribution of Late Neolithic sites is comparable with the Early Neolithic (Fig 8D).

#### 4.3.3 Site Exploitation Territories (SET) and settlement dynamics

**Fig 9:**
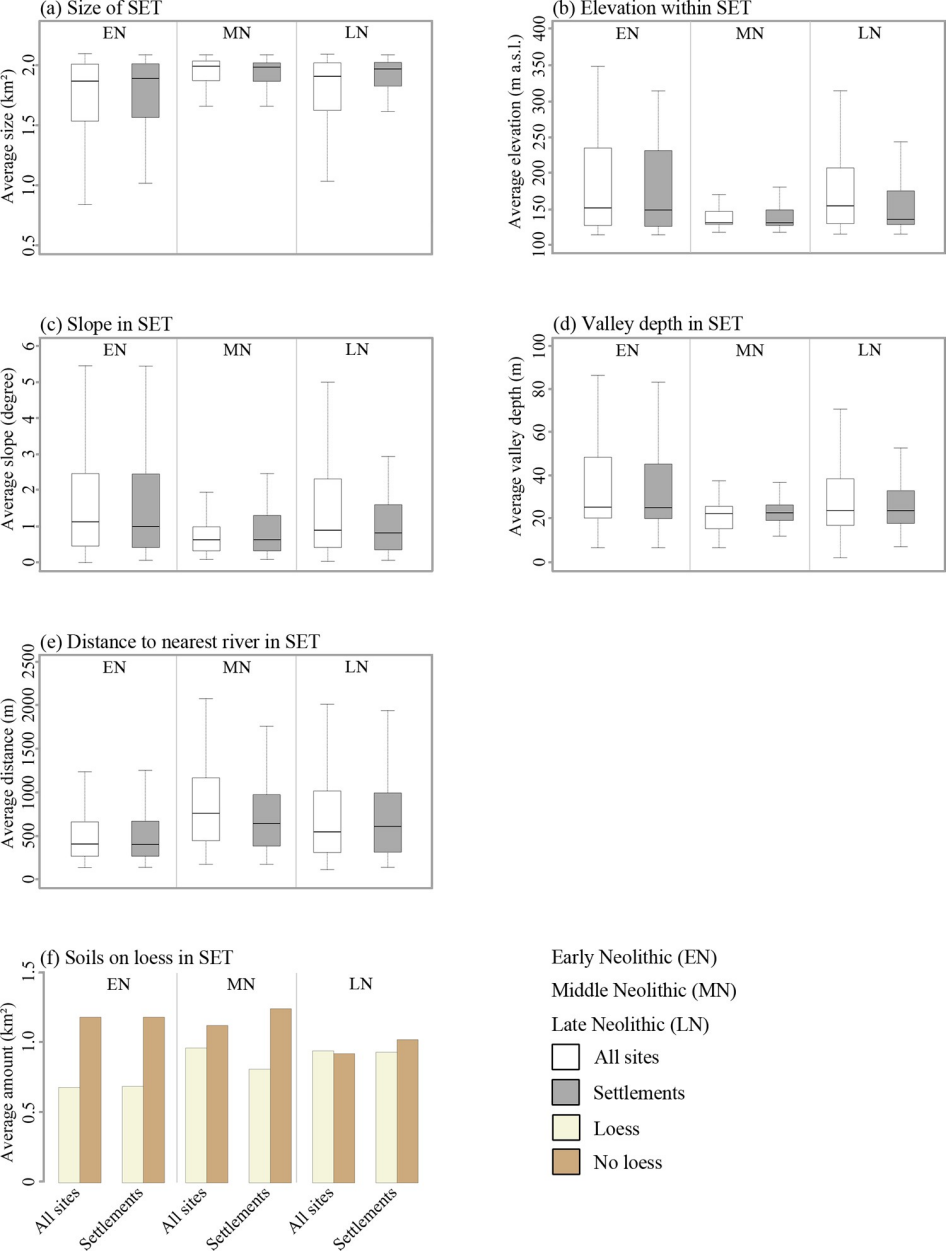
Analyses of site exploitation territories. (a) Size of modelled SET, (b) terrain elevation, (c) slope, (d) valley depth, (e) river distance and (f) soils on loess and. For more information on each terrain covariate see S1 Table.

For all sites, a differentiation of the Neolithic periods is possible based on the smallest SETs observed. For the Early and Late Neolithic sites very small minimal SETs with sizes of about 0.7 km^2^ were identified. In contrast, the smallest SETs for Middle Neolithic sites cover areas of 1 km^2^. The same pattern is true for the settlements of the three periods (Fig 9A).

While the immediate surroundings of Early Neolithic sites are on average located at an elevation of 182 m a.s.l., the SETs of Middle Neolithic sites are characterised by lower elevations (141 m a.s.l.). Late Neolithic SETs are characterised by elevations similar to those determined for the Early Neolithic sites (176 m a.s.l.). A separate evaluation of settlement sites results in comparable values for the Early and Middle Neolithic, i.e. 175 and 143 m a.s.l. (Fig 9B). In contrast, the average elevation within the SETs of Late Neolithic settlements is similar to that of Middle Neolithic settlements (157 m a.s.l.).

With regard to slope, differences between the Early and Late Neolithic on the one hand (ca. 1.6°), and the Middle Neolithic on the other hand (ca. 0.8°) can be observed. In addition, the average value for Late Neolithic settlement SETs (1.2°) is lower than the average for all sites from this period (Fig 9C).

By calculating the average valley depth within the SETs for all sites, the Early and Late Neolithic (ca. 38 and 35 m) can be distinguished from the Middle Neolithic (27 m). However, the average valley depth in the vicinity of Early and Late Neolithic settlements is slightly below the average for all sites for the respective periods. The opposite is true for the Middle Neolithic (Fig 9D).

For all sites, an increased river distance of about 450 m can be observed with the transition from the Early Neolithic (about 520 m) to the Middle Neolithic (980 m). For the Late Neolithic, the average river distance decreased to about 820 m. The values for Early and Late Neolithic settlements did not differ from those observed for all sites from the respective periods. In contrast, the average river distance within SETs of Middle Neolithic settlements is approx. 100 m lower compared to all sites from that period (Fig 9E).

The descriptive statistics of the proportion of loess-soils in the SETs did not enable any differentiation between the Neolithic periods (Fig 9F).

## 5 Discussion

### 5.1 Spatial and chronological representativeness of the Neolithic record

#### 5.1.1 Site distribution with respect to modern land use and archaeological surveys

Modern land use classes are not tied to archaeological criteria. Therefore, these represent an independent sample with regard to the distribution of archaeological sites in both spatial and chronological terms [100]. The analysis of the CLC dataset showed that urban areas, arable land and open-cast mines are positive filters, since the surface is frequently opened up in these areas by various activities such as construction measures, ploughing or mining. This often leads to the discovery of buried sites (Table 2). In contrast, forest and grassland were identified as negative filters, since they reduce the likelihood of discovering new sites due to dense vegetation and little to no surface disturbances. These negative filters only dominate in the higher altitudes of the southern study area. It is important to acknowledge the widespread distribution of positive filters especially in the Central Uplands, where only few Neolithic sites have been discovered (Fig 7).

Archaeological surveys led to an increase in Neolithic site densities between Zeitz and Gera (Fig 8). However, this only applies to the Early and Late Neolithic. In contrast, occasional surveys in the Central Uplands did not lead to the discovery of Neolithic sites in those areas. The use of aerial photography did not affect the Neolithic site distributions.

#### 5.1.2 Site distribution with respect to archaeological site visibility and preservation of material remains

Kappa scores of our RF analyses between 0.53 and 0.59 were found for the depth of Neolithic sites, as well as for the presence/absence and depth of Neolithic pottery with respect to selected topographic, climatic, soil physical and geochemical terrain covariates (S6–S8 Tables). These Kappa scores indicate a substantial agreement between the models and the archaeological classifications [125, 126]. This means that the visibility of Neolithic sites and the distribution of material groups as shown in Fig 6 were influenced by geomorphological terrain dynamics and weathering. In this context, the most important terrain covariates were elevation–and subsequently correlated climate parameters such as temperature, frost, precipitation etc.–, slope and the proportion of coarse fragments in soils that largely control/are partly controlled by soil erosion and accumulation processes, and copper distribution that is an unspecific proxy for climatic, geological and pedological factors as well as agricultural activities and land cover [168, 169]. The only exception is the distribution of stone tools with regard to their depth. Here, the low Kappa score of 0.21 indicates poor agreement (S9 Table), meaning that the applied terrain covariates hardly describe the distribution of (non-)buried stone tools. Consequently, the preservation conditions for stone tools are similar in the Central Uplands and in the southernmost part of the Northern German Plain, since these were not significantly influenced by the geomorphological terrain dynamics. This finding is important, since stone tools occur at basically every Neolithic site. Hence, the distribution of stone tools can be regarded as a reliable proxy for areas that were inhabited during the Neolithic. Furthermore, our RF results show that despite the general plausibility of the chosen accuracy measurements that were based on general assumptions, the parallel application of several quality measurements is necessary. This is caused by their different specifics according to the data conditions. Here, this is demonstrated by the analysis of the distribution of stone tools with regard to their depth, since error rate, Precision, Recall and the F_1_-measure were in line with all previous measurements and showed a positive agreement, whereas this was opposed by the low Kappa value. The influence of the unbalanced class distributions is evident here in the quality measures (Table 1).

Our finding that the lack of pottery in the Central Uplands (Fig 6B) possibly results from poor local preservation conditions confirms previous speculations for the study area [170–172]. However, also subsistence strategies must be taken into account for this phenomenon, as well as for the generally low site densities in the Central Uplands that are a well-known phenomenon in Europe [173]. In contrast to the lowlands, the economic use of low mountain ranges was probably limited to transhumance, which tends to leave few archaeological traces due to its seasonal character and smaller groups of people compared to sedentary farming communities. This mobile way of life meant that only the most necessary equipment could be taken along. Consequently, the pottery carried along also had to be reduced to a minimum [174, 175]. Therefore, the absence of Neolithic pottery in the Central Uplands has to be considered with respect to (I) the context of local preservation conditions, and (II) an economic strategy that leaves very little pottery behind and may have been practised in these landscapes. However, further research is be needed to decide which of these two factors has a greater impact on the Neolithic record in the study area.

#### 5.1.3 Neolithic floodplain sites

Neolithic finds are also known from the sediments of the Weiße Elster floodplain [176]. Originally, these Neolithic sites were most likely located on terrace islands or terrace peninsulas that later became part of the floodplain as we see it today [177, 178]. However archaeological, pedological and chronological investigations [179, 180] demonstrated that the Neolithic floodplain sites are covered by Subboreal to Sub-Atlantic silt-clay overbank fines (floodloam). As a consequence, the site density in these areas may be somewhat higher than current archaeological maps indicate [56]. Similarly, missing knowledge about prehistoric settlement patterns due to thick coverages of prehistoric settlements with younger floodloam are also known from other floodplains [181–183].

In a nutshell, based on an assessment of former field surveys, CLC and RF analyses, we can confirm that apart from the distribution of Neolithic pottery and sites in floodplain areas the site distribution in the study area reflects the Neolithic settlement dynamics quite well.

#### 5.1.4 Chronological distribution of Neolithic sites

The uneven distribution of settlements, burial sites, and stray finds over the different Neolithic periods indicates that their archaeological identification varies between these periods (S3 and S5 Tables). Consequently, these varying likelihoods of identifying different site categories may be one of the driving factors behind fluctuations in site frequencies (Table 3). For example, Middle Neolithic sites might be under-represented because they were identified based on pottery that is rarely decorated. To do so, the shape of largely complete vessels has to be considered for their archaeological dating [184–189]. Hence, Middle Neolithic pottery can easily be overlooked as soon as vessel shapes can no longer be derived from pottery fragments [41, 56, 57]. In contrast, Early and Late Neolithic pottery is decorated with characteristic patterns, and therefore easy to identify [187–189]. Furthermore, these latter periods are associated with distinctive stone tools: While shoe-last celts (German: Schuhleistenkeil) are typical for the Early Neolithic, faceted axes (German: Facettenaxt) belong to the most characteristic Late Neolithic artifacts [188, 189]. Therefore, the proportion of stray finds is remarkably high for these periods and small for the Middle Neolithic (S3 Table). Moreover, the ratio between settlements and burial sites varies for each period, e.g., more settlements than burial sites are known from the Early and Middle Neolithic while the opposite is true for the Late Neolithic (S3 Table). This is due to the fact that shallow graves were common during the former periods, while the deceased were buried in mounds during the Late Neolithic [89, 90, 190]. In contrast to burial mounds, shallow graves are hardly visible on the surface and therefore discovered usually by chance [108, 135, 191, 192]. Furthermore, the archaeological identification of Late Neolithic houses is difficult due to their construction techniques [90, 99, 193]. Results from excavations in Profen [193], Seifartsdorf [194] and Lucka [195, 196] indicate that Late Neolithic settlements and burial mounds usually exist in close proximity to each other. Accordingly, Late Neolithic settlements are probably under-represented in the study area, especially in the south where mainly stray finds and burials have been discovered so far. After all, various reasons could have caused the comparatively small number of Middle Neolithic settlements: In addition to a real reduction of settlement activities during that period, this also includes the difficulties in identifying pottery from this period and also the fact that Middle Neolithic settlements were somewhat smaller than settlements from the Early or Late Neolithic. Moreover, the associated features (houses, pits, wells, etc.) are often only loosely scattered. Therefore, Middle Neolithic settlements are most likely to be discovered during excavations of large contiguous areas. In our study area, this was illustrated during excavations at Droßdorf [158, 197], Großdalzig [198] and Zauschwitz [199]. Because all the discussed aspects are inherent to the Neolithic periods themselves, these phenomena were observed archaeologically everywhere in Central Germany [33, 41, 56, 57, 101, 171, 200–204]. Finally, to assess whether the observed reduction of Middle Neolithic compared with Early and Late Neolithic finds was real or not, independent archives of former settlement activity would be necessary for comparisons with our settlement record. However, so far only few pollen records with very limited stratigraphical and chronological resolution exist in this region for the Neolithic period [101, 172, 205–208], so that such comparisons cannot be made so far.

**Table 3:** Average Neolithic site frequencies per 250 km^2^ in the study area and adjacent landscapes.

Neolithic period	Erfurt	Downstream section of the Bode river	North-west Saxony	Lake Göttwitz	Gothaer Land	Weisse Elster	Elbe-Saale Region	Dresden Elbe valley	Former district of Riesa-Großenhain	Dresden Elbe vally And lower part of eastern Ore Mountains	Ore Mountains
Early Neolithic, 5500–4400 cal. BCE	10,2	3,6	4,7	3,4	3,5	2,7	1,4	3,0	2,0	1,9	0,7
Middle Neolithic, 4400–2800 cal. BCE	2,7	1,6	1,7	0,5	0,3	0,4	1,0	0,4	0,3	0,0	0,2
Late Neolithic, 2800–2300 cal. BCE	15,0	10,4	6,8	6,2	3,4	4,9	4,3	1,9	1,8	1,6	0,9
Area	ca. 120 km^2^	ca. 245 km^2^	250 km^2^	430 km^2^	800 km^2^	3026 km^2^	4600 km^2^	374 km^2^	822 km^2^	400 km^2^	ca. 3300 km^2^
Reference	Walter et al. 1987	Kaufmann 1967	Wegener 2014	Hilbig 1993	Müller 1980	present study	Starling 1983	de Vries 2013	Balfanz 2003	Jacob 1982	Christl 2004

The Vogtland has not been included, because the respective study does not provide concrete data with respect to the number of Neolithic sites discovered in the area [171].

### 5.2 Local settlement dynamics and their regional context

**Fig 10:**
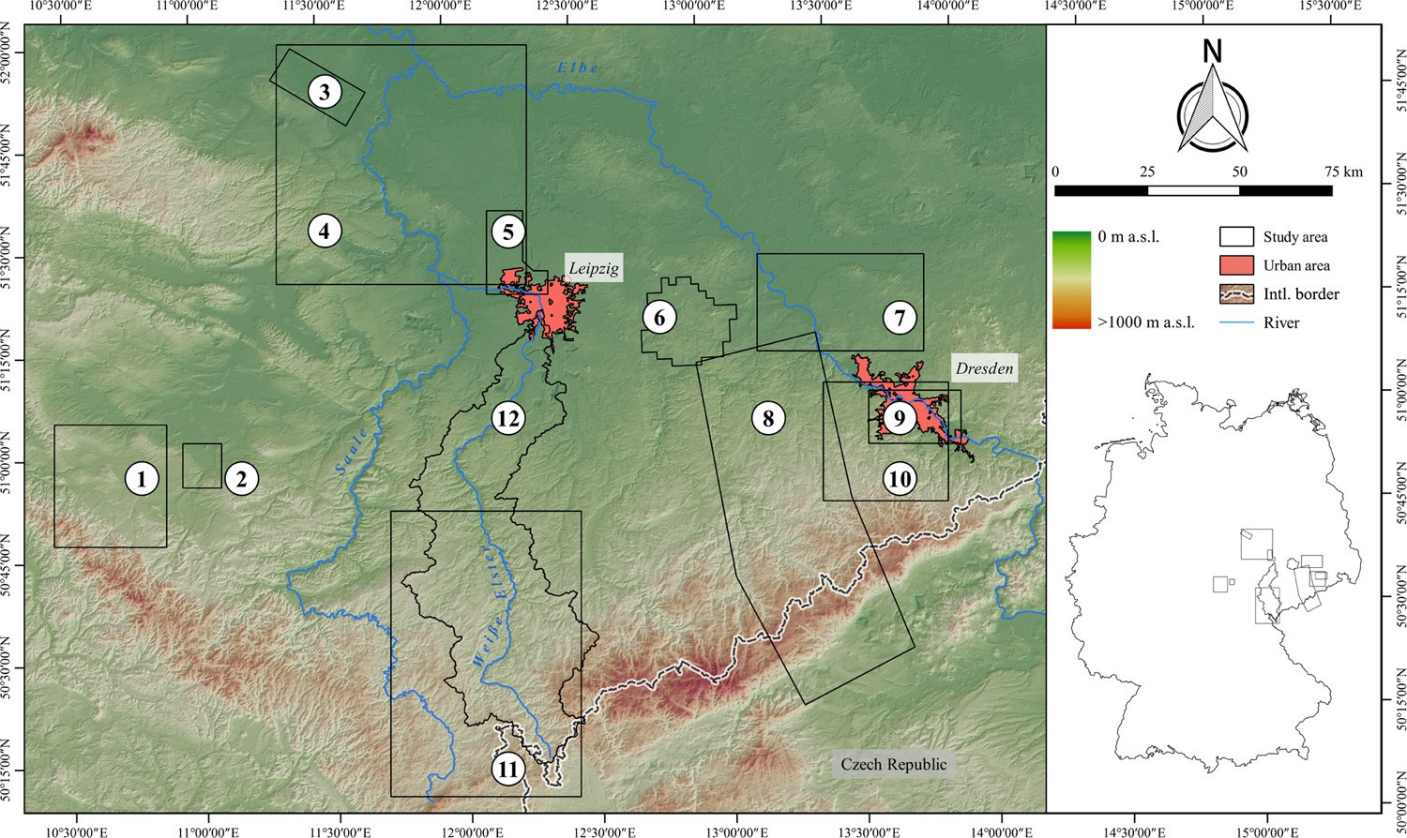
Study areas in Central Germany mentioned in the text and in Table 3. (1) Gothaer Land [33], (2) Erfurt [200], (3) downstream section of the Bode river [201], (4) Elbe-Saale-Region [202], (5) North-West Saxony [57], (6) Lake Göttwitz [101], (7) former district of Riesa-Großenhain [56], (8) Ore Mountains [203], (9) Dresden Elbe valley [41], (10) Dresden Elbe valley and lower part of eastern Ore Mountains [204], (11) Vogtland [171], (12) catchment of the Weiße Elster river (this study).

The Neolithic settlement dynamics in the catchment of the river Weiße Elster largely match findings from neighbouring landscapes, including the distinctive decline in Middle Neolithic site frequencies (Table 3; Fig 10). Furthermore, it was repeatedly observed across Central Germany that more elevated zones, which were less favourable for agriculture use were inhabited during the Early and Late Neolithic, but were largely abandoned during the Middle Neolithic (see references in Fig 10).

These settlement dynamics are reflected in the results of our SET analyses. With respect to elevation show that the average elevation values within the SETs dropped at the transition from the Early to the Middle Neolithic. Although no other landscape was analysed using SET and former statistical approaches were based on point coordinates and smaller datasets (see references in Fig 10), our results agree with observations from other studies [33, 57, 203]. Furthermore, our SET analyses point to greater average distances of Middle and Late Neolithic settlements to rivers compared to the Early Neolithic, which is in line with former observations [33, 57, 209]. However, site distributions with regard to valley depth were not carried out during former studies so far. Accordingly, there is no information whether the dynamics observed in our study area can also be seen elsewhere. In addition, our SET analyses illustrate that Neolithic settlement dynamics were not linked with different proportions of soils on loess. This is in line with observations from adjacent study areas in Central Germany [57, 101].

Another common feature with other regional studies is the concentration of the highest site densities mostly along the floodplains of large rivers (Fig 8; S1 Fig) [23, 210]. This phenomenon has been discussed against the background of the distribution of forests and open land, although there are no pollen records available that can be used to reconstruct the Neolithic vegetation coverage in Central Germany in detail [56]. Hypothetical mappings of forest and open land have been prepared using medieval records, onomastic data and soil maps. Based on these studies, a forest coverage is assumed for the Neolithic floodplains [210–213]. In our study area, this is supported by tree logs especially of *Quercus*, but also *Fraxinus*, *Alnus*, *Pinus* and *Ulmus* with Neolithic ages in floodplain sediments of the lower Weiße Elster valley [214, 215]. In the northern part of our study area the forest is supposed to haven been contrasted with the adjacent loess areas, for which a thin and patchy forest vegetation is assumed [56, 97, 210, 216]. Therefore, the margins between the floodplains and the neighbouring loess-covered slopes may have been preferred settlement areas due to economic benefits, since they offered easy access to both fertile soils and adjacent forests. The latter could be exploited for the extraction of wood for the construction of houses and wells, or be used as fuel, hunting grounds or for forest grazing by cattle, sheep, goats and pigs. The rivers themselves might have been used for fishing and fresh water supply [23, 210, 217, 218]. Especially in the northern part of the study area fresh water resources were probably rare outside the main river valleys. This could have been caused by the relatively flat topography, leading to an extensive lack of small tributary valleys that potentially offer surface water resources by small creeks and sources. Furthermore, the climate of central Europe before ca. 5 ka was generally drier compared with today [5], so that several small current rivers were dry during that period [106]. Altogether, this suggests that these multi-purpose near-river locations were probably preferred settlement areas to compensate economic stresses in these environments (Fig 8) [210]. Recently, this traditional explanation was somehow challenged by large-scale excavations in our study area, during which burial sites and settlements were discovered in the watershed areas between the rivers Weiße Elster and Saale [193] and between the rivers Weiße Elster and Pleiße [219, 220]. However, Neolithic wells were discovered in both, the large river valleys and those regions far away from the large valleys. In the latter areas, these, could have helped to provide water resources for the Neolithic settlers and therefore facilitated settlement activities [220, 221].

### 5.3 Driving factors of the local settlement dynamics

The main drivers of Neolithic settlement dynamics are still being debated. Among other things, it has been hypothesised that strong population growth during the Early and Late Neolithic may have contributed to the settlement on lower-yielding soils in the Central Uplands [170, 201, 202, 222]. In addition, a connection with climatic conditions has been considered: A generally drier climate during the Early and Late Neolithic could have been compensated by settlement activities in the more humid Central Uplands and well constructions in the southernmost part of the Northern German Plain [223, 224]. In fact, most Neolithic wells in the study area have been dated to the Early or Late Neolithic using dendrochronology and radiocarbon dating [158]. Finally, the concentration of Middle Neolithic settlement on the lowlands in the southernmost part of the Northern German Plain was interpreted as an indication that the comparatively high annual temperatures and corresponding longer vegetation periods in this landscape were of special importance for the applied subsistence strategies [99].

## 6 Conclusion

Based on a dataset of 1365 Neolithic sites that was compiled in the frame of a joint geoarchaeological research project in Central Germany, we combined methods of archaeological source criticism with machine learning to discuss the large-scale Neolithic settlement dynamics in the immediate catchment of the Weiße Elster river in Central Germany. Our research lead to the following conclusions:

- The use of local area files (German: Ortsakten) proved to be extremely valuable. It turned out that only half of the actually existing sites have been published so far.
- Analyses of site distributions with respect to modern land use and field surveys indicate that the basic trends of Neolithic settlement dynamics can be derived from the recorded sites. However, by investigating the influence of terrain covariates on the depth of sites using the Random Forests algorithm, we demonstrate that the visibility of Neolithic sites is influenced by geomorphological terrain dynamics: While the majority of the sites is buried in the southernmost part of the Northern German Plain, the opposite is true for the Central Uplands. Furthermore, Random Forests algorithm is able to reproduce the distribution of sites with(-out) pottery. These results can be explained in different ways: Firstly, the preservation of pottery in the Central Uplands may be not as good as in the southernmost part of the Northern German Plain. Secondly, the low amount of Neolithic pottery in the Central Uplands may also be a result of economic strategies that leave very little pottery behind in general. Nevertheless, the Random Forests algorithm offers new opportunities for archaeological source criticism, which is crucial for identifying potential biases in large archaeological datasets and deriving settlement dynamics.
- The distribution of Early Neolithic sites in the lowlands of the southernmost part of the Northern German Plain and the mountainous landscape between Zeitz and Gera is characterized by clusters along the Weiße Elster River. In contrast, the geographic focus of Middle Neolithic land use was restricted to the northern lowlands, whereas the Late Neolithic site distribution pattern resembled that of the Early Neolithic. The site frequencies are marked by a decline in the Middle Neolithic, and a re-increase during the Late Neolithic. These observed trends could at least partly be consequences of diachronically different properties of the archaeological record.
- In the southernmost part of the Northern German Plain, Neolithic land use generally took place at the transition between the floodplain of the Weiße Elster River and loess-covered soils outside the valley. These settlement patterns are common in Central Germany, and were explained with environmental economic factors. However, both settlements and burial sites have been discovered recently in the watershed areas between Weiße Elster and Saale River as well as between the rivers Weiße Elster and Pleiße. Accordingly, the low site densities in greater distances from rivers might be a reflection of a lack of research.
- Analyses of SETs essentially reflect the settlement dynamics between the southernmost part of the Northern German Plain and the Central Uplands. Early Neolithic settlements were located closer to river valleys, and were situated in relatively elevated areas above the river valleys. In contrast, settlements dating to later periods were not located as high above the river valleys, but in larger distances from the latter. However, Neolithic settlement dynamics were not linked with different proportions of soils on loess.
- We identified potential for future research in the region: Numerous Neolithic sites in the surroundings of Gera could not be assigned to any period so far, but might be re-dated by means of archaeological methods. Moreover, the Late Neolithic in the Central Uplands is mainly known through burials and stray finds.

Generally, our study demonstrates the high value of systematic studies of diachronic archaeological settlement patterns to understand varying regional human activity in space and time. However, since such records can show different properties and sensitivities through time, complementary studies of other environmental or historic archives documenting regional human impact could help to identify possible intrinsic biases. In the future complementary environmental archives will be analysed to get deeper insights into early settlement processes and their environmental background. A key question than will be to which degree human activities and climatic factors were responsible for fluvial soil deposits.

## Supporting information

S1 Table Terrain covariates. (XLSX)

S2 Table Topographic maps 1:25000 used to modify topography and river data. (XLSX)

S3 Table Neolithic sites with respect to the accuracy of their location and site functions. (XLSX)

S4 Table Neolithic sites with respect to the status of their publication. (XLSX)

S5 Table Proportion of (non-)intentional as well as unknown modes of discovery associated with each Neolithic period. (XLSX)

S6 Table Results of random forests analyses with respect to interrelation of terrain covariates and site depth. (XLSX)

S7 Table Results of random forests analyses with respect to interrelation of terrain covariates and the distribution of material groups. (XLSX)

S8 Table Results of random forests analyses with respect to interrelation of terrain covariates and the distribution of (non-)buried pottery. (XLSX)

S9 Table Results of random forests analyses with respect to interrelation of terrain covariates and the distribution of (non-)buried stone tools. (XLSX)

S1 Fig Study area with respect to geomorphographic units of Germany. GMK1000RV2.0, (C) BGR, Hannover, 2006. Translated by Jan Miera.
(TIF)

S2 Fig Visualization of changes performed on the local topography. DEM data were provided by the SRTM Digital Elevation Database version 4.1 (Available from: http://srtm.csi.cgiar.org).
(TIF)

S3 Fig Changes performed on the local river network. River data were provided by the Saxon State Office for Environment, Agriculture and Geology, the Saxony-Anhalt State Agency for Flood Protection and Water Management and the Thuringian State Office for Soil Management and Geoinformation.
(TIF)

S4 Fig Correlation of terrain covariates. Here, we used R package *corrplot* from Wei T, Simko V. R package ’corrplot’: Visualization of a Correlation Matrix. (Version 0.92). 2021 [Cited 2022 February 1]. Available from: https://github.com/taiyun/corrplot.
(TIF)

S1 Dataset Neolithic sites in the Weiße Elster river catchment. This table contains all Neolithic sites from our study area and can be used to reproduce our results with regard to the status of research.
(TXT)

S2 Dataset Random forests analyses. This table can be used to reproduce our Random Forests analyses.
(TXT)

S3 Dataset Site Exploitation Territories (SET). This table can be used to reproduce our SET analyses.
(TXT)

S1 References References cited only in the supporting information. (DOCX)

## References

[1] Cendrero A, Remondo J, Bonachea J, Rivas V, Soto J. Sensitivity of landscape evolution and geomorphic processes to direct and indirect human influence. Geografia Fisica e Dinamica Quaternaria. 2006; 29:125–137.

[2] Dotterweich M. The history of soil erosion and fluvial deposits in small catchments of central Europe: Deciphering the long-term interaction between humans and the environment—a review. Geomorphology. 2008; 101:192–208. 10.1016/j.geomorph.2008.05.023

[3] Brown AG, Tooth S, Bullard JE, Thomas DSG, Chiverell RC, Plater AJ, et al. The geomorphology of the Anthropocene: emergence, status and implications. Earth Surf Process Landf. 2017; 42:71–90. 10.1002/esp.3943

[4] Menges J, Hovius N, Andermann C, Dietze M, Sowboda C, Cook CL, et al. 2019. Late Holocene landscape collapse of a trans-Himalayan dryland: Human impact and aridification. Geophys Res Lett. 2019; 46:13814–13824. 10.1029/2019GL084192

[5] Litt T, Schölzel C, Kühl N, Brauer A. Vegetation and climate history in the Westeifel Volcanic Field (Germany) during the past 11 000 years based on annually laminated lacustrine maar sediments. Boreas. 2009; 38:679–690. 10.1111/j.1502-3885.2009.00096.x

[6] Broothaerts N, Verstraeten G, Kasse C, Bohncke S, Notebaert B, Vandenberghe J. 2014. Reconstruction and semi-quantification of human impact in the Dijle catchment, central Belgium: a palynological and statistical approach. Quat Sci Rev. 2014; 102:96–110.

[7] Argiriadis E, Battistel D, McWethy DB, Vecchiato M, Kirchgeorg T, Kehrwald NM, et al. Lake sediment fecal and biomass burning biomarkers provide direct evidence for prehistoric human-lit fires in New Zealand. Scientific Reports. 2018; 8:12113. doi: 10.1038/s41598-018-30606-3 30108240 PMC6092367

[8] Suchodoletz H von, Tinapp C, Lauer T, Glaser B, Stäuble H, Kühn P, Zielhofer C. Distribution of Chernozems and Phaeozems in Central Germany during the Neolithic period. Quat Int. 2019; 511:166–184. 10.1016/j.quaint.2017.10.041

[9] Schier W. Die vorgeschichtliche Besiedlung im südlichen Maindreieck, Teil 1: Text. Materialhefte zur bayerischen Vorgeschichte. Reihe A–Fundinventare und Ausgrabungsbefunde 60,1. Kallmünz/Opf: Lassleben; 1990.

[10] Pankau C. Die Besiedlungsgeschichte des Brenz-Kocher-Tals (östliche Schwäbische Alb) vom Neolithikum bis zur Latènezeit, Teil I. Universitätsforschungen zur prähistorischen Archäologie 142,1. Bonn: Habelt; 2007.

[11] Fender P. Bayern in der Vorgeschichte–Eine GIS-gestützte Analyse der Siedlungslandschaft und der Einsatz von Open Data in der Archäologie. Marburg: Philipps-Universität Marburg; 2017. 10.17192/z2017.0774.

[12] Miera JJ. Ur- und frühgeschichtliche Siedlungsdynamiken zwischen Gunst- und Ungunsträumen in Südwestdeutschland–Landschaftsarchäologische Untersuchungen zur Baar und den angrenzenden Naturräumen des Schwarzwaldes und der Schwäbischen Alb. RessourcenKulturen 10. Tübingen: Tübingen University Press; 2020. 10.15496/publikation-45820

[13] Tode A. Organisation und praktische Durchführung einer allgemeinen archäologischen Lan-desaufnahme. Vorgeschichtliches Jahrbuch, 1926; 3:10–21.

[14] Gummel H. Forschungsgeschichte in Deutschland. Die Urgeschichtsforschung und ihre historische Entwicklung in den Kulturstaaten der Erde 1. Berlin: De Gruyter; 1938.

[15] Schirnig H. Einige Bemerkungen zur archäologischen Landesaufnahme. Nachrichten aus Niedersachsens Urgeschichte, 1966; 35:3–13.

[16] Stoll H. Urgeschichte des Oberen Gäues. Veröffentlichungen des Württembergischen Landesamts für Denkmalpflege 7. Öhringen: Rau; 1933.

[17] Fischer E. Beiträge zur Kulturgeographie der Baar. Badische Geographische Abhandlungen 16. Freiburg im Breisgau: Waibel; 1936.

[18] Gradmann R. Das ländliche Siedlungswesen des Königreichs Württemberg. Forschungen zur deutschen Landes- und Volkskunde. 1917; 21:1–136.

[19] Deecke W. Geologisch-morphologische Bemerkungen zur Prähistorie Badens. Praehistorische Zeitschrift. 1918; 10:40–57. 10.1515/prhz.1918.10.1.40

[20] Brunnacker K, Kossack G. Ein Beitrag zur vorrömischen Besiedlungsgeschichte des niederbayerischen Gäubodens. Archaeologia Geographica. Beiträge zur vergleichenden geographisch-kartographischen Methode in der Urgeschichtsforschung. 1956; 5/6:43–54.

[21] Sielmann B. Der Einfluß der Umwelt auf die neolithische Besiedlung Südwestdeutschlands unter besonderer Berücksichtigung der Verhältnisse am nördlichen Oberrhein, Acta Prähistorica et Archaeologica. 1971; 2:65–197.

[22] Linke W. Frühestes Bauerntum und geographische Umwelt. Eine historisch-geographische Untersuchung des Früh- und Mittelneolithikums westfälischer und nordhessischer Bördenlandschaften. Bochumer geographische Arbeiten 28. Paderborn: Schöningh; 1976.

[23] Sielmann B. Der Einfluß der geographischen Umwelt auf die linien- und stichbandkeramische Besiedlung Mitteldeutschlands. Jahresschrift für mitteldeutsche Vorgeschichte. 1976; 60:305–329.

[24] Jankuhn H. Einführung in die Siedlungsarchäologie. Berlin: De Gruyter; 1977.

[25] Linke W. Die Reichsbodenschätzung als Hilfsmittel der Prähistorie. Praehistorische Zeitschrift. 1979; 54:177–186.

[26] Knopf T. ‚Umwelt‘ als Forschungsgegenstand. Konzepte und Theorien. In: Eggert MKH, Veit U, editors. Theorie in der Archäologie. Zur jüngeren Diskussion in Deutschland. Tübinger Archäologische Taschenbücher 10. Tübingen: Waxmann; 2013. pp. 63–99.

[27] Müller-Scheeßel N. Mensch und Raum: Heutige Theorien und ihre Anwendung. In: Eggert MKH, Veit U, editors. Theorie in der Archäologie: Zur jüngeren Diskussion in Deutschland. Tübinger Archäologische Taschenbücher 10. Münster: Waxmann; 2013. pp. 101–137.

[28] Miera JJ. in Press. Ein ideengeschichtlicher Überblick zum Umgang mit Gunst und Ungunst in der Prähistorischen Archäologie. In: Miera JJ, Knopf T, Scholten T, Kühn P., editors. Gunst/Ungunst: Nutzung und Wahrnehmung von (Marginal-)Räumen. Tübingen: Tübingen University Press; Forthcoming.

[29] Gebhard K. Die vorgeschichtliche Besiedlung des Kreises Groß-Gerau. Materialien zur Vor- und Frühgeschichte von Hessen 25. Wiesbaden: Landesamt für Denkmalpflege Hessen; 2007.

[30] Heun S. Besiedlungsgeschichte der Latènezeit am Beispiel des Landkreises Offenbach. Siedlungsgeschichtliche Auswertung von Altfunden und neuen Fundstellen im Hinblick auf Kontinuitätsfragen. Marburg: Philipps Universität Marburg; 1999. 10.17192/z2004.0519

[31] Ickler S. Bronze- und Eisenzeitliche Besiedlung im Stadtgebiet von Krefeld, mittlerer Niederrhein. Köln: Universität zu Köln; 2007 [Cited 2021 November 24]. Available from: http://kups.ub.uni-koeln.de/id/eprint/3163

[32] Mischka D. Methodische Aspekte zur Rekonstruktion prähistorischer Siedlungen. Landschaftsgenese vom Ende des Neolithikums bis zur Eisenzeit im Gebiet des südlichen Oberrheins. Freiburger archäologische Studien 5. Rahden/Westfalen: Leidorf; 2007.

[33] Müller DW. Die ur- und frühgeschichtliche Besiedlung des Gothaer Landes: Naturräumliche Voraussetzungen und Kulturenfolge. Alt Thuring. 1980; 17:19–180.

[34] Schmotz K. Die vorgeschichtliche Besiedlung im Isarmündungsgebiet. Materialhefte zur Bayerischen Vorgeschichte, Reihe A–Fundinventare und Ausgrabungsbefunde 58. Kallmünz/Opf: Lassleben; 1989.

[35] Bevan A, Lake M, editors. Computational approaches to archaeological spaces. Walnut Creek: Left Coast Press; 2013.

[36] Gillings M, Hacigüzeller P, Lock GR, editors. Archaeological spatial analysis: a methodological guide. London: Routledge; 2020.

[37] Nakoinz O, Knitter D. Modelling human behaviour in landscapes: basic concepts and modelling elements. Quantitative Archaeology and Archaeological Modelling 1. Heidelberg: Springer Nature; 2016.

[38] Saile T. Relief Intensity and the Formation of the Archaeological Record. In: Gaul W, Ritter G., editors. Classification, Automation, and New Media. Studies in Classification, Data Analysis, and Knowledge Organization. Berlin: Springer; 2002. pp. 479–489. 10.1007/978-3-642-55991-4_52

[39] Miera JJ, Henkner J, Schmidt K, Fuchs M, Scholten T, Kühn P, Knopf T. Neolithic settlement dynamics derived from archaeological data and colluvial deposits between the Baar region and the adjacent low mountain ranges, southwest Germany. E & G Quaternary Science Journal. 2019; 68:75–93. 10.5194/egqsj-68-75-2019

[40] Meylemans E, Poesen J, In ’t Ven I, editors. The Archaeology of Erosion, the Erosion of Archaeology. Proceedings of the Brussels Conference, april 28–30 2008. Relicta Monografieën 9. Brussel: Flanders Heritage Agency, 2014.

[41] de Vries P. Prähistorische Siedlungsplatzwahl in der Dresdner Elbtalweitung. Veröffentlichungen des Landesamtes für Archäologie 58. Dresden: Landesamt für Archäologie; 2013.

[42] Sauer F. Spätpaläolithische Landnutzungsmuster in Bayern. Nürnberg: Friedrich-Alexander-Universität Erlangen-Nürnberg; 2018. urn:nbn:de:bvb:29-opus4-92875

[43] Polla S, Verhagen P, editors. Computational approaches to the study of movement in archaeology: theory, practice and interpretation of factors and effects of long term landscape formation and transformation. Berlin: De Gruyter; 2014. 10.1515/9783110288384

[44] Roper DC. The Method and Theory of Site Catchment Analysis. A Review. Advances in Archaeological Method and Theory. 1979; 2:119–140.

[45] Dennell R. The Use, Abuse and Potential of Site Catchment Analysis. In: Findlow FJ, Ericson JE, editors. Catchment Analysis. Essays on Prehistoric Resource Space. Anthropology UCLA 10. Los Angeles: Department of Anthropology, University of California; 1980. pp. 1–20.

[46] Tiffany JA, Abbott LR. Site Catchment Analysis. Application to Iowa Archaeology. J Field Archaeol. 1982; 9:313–322. 10.1179/009346982791504634

[47] Gringmuth-Dallmer E, Altermann M. Zum Boden als Standortfaktor ur- und frühgeschichtlicher Siedlungen. Jahresschrift für mitteldeutsche Vorgeschichte. 1985; 68:339–355.

[48] Paetzold D. Bemerkungen zum Siedlungsverhalten neolithischer bis latènezeitlicher Bevölkerungen zwischen Regensburg und Deggendorf. Gibt es Besiedlungsschwerpunkte in Abhängigkeit von naturräumlicher Gliederung und Bodenbeschaffenheit? Bayerische Vorgeschichtsblätter. 1992; 57:77–102.

[49] Cappenberg K. Landscape as a Feature: Using GIS and Statistics to Compare Two Types of Early Neolithic Sites in Lesser Poland. In: Kienlin TL, Valde-Nowak P, Korczyńska M, Cappenberg K, Ociepka J, editors. Settlement, communication and exchange around the Western Carpathians: International Workshop held at the Institute of Archaeology, Jagiellonian University, Kraków, October 27–28th, 2012. Oxford: Archaeopress; 2014. pp. 51–66.

[50] Henry DO, Belmaker M, Bergin SM. The effect of terrain on Neanderthal ecology in the Levant. Quat Int. 2017; 435:94–105. 10.1016/j.quaint.2015.10.023

[51] Bailey GN, Davidson I. Site Exploitation Territory and Topography. Two Case Studies from Palaeolithic Spain. J Archaeol Sci. 1983; 10:87–115. 10.1016/0305-4403(83)90044-4

[52] Schier W. Zur vorrömischen Besiedlung des Donautales südöstlich von Regensburg. Bayerische Vorgeschichtsblätter. 1985; 50:9–80.

[53] Sorcan BJ. Die Vorgeschichtliche Besiedlung des Unteren Altmühltales. Arbeiten zur Archäologie Süddeutschlands 26. Büchenbach: Faustus; 2011.

[54] Pfister D. Vor- und frühgeschichtliche Besiedelung im östlichen Unterfranken von der ältesten Linienbandkeramik bis zum Ende der römischen Kaiserzeit. Würzburg: Universität Würzburg; 2014. urn:nbn:de:bvb:20-opus-105521

[55] Obst R. Die Besiedlungsgeschichte am nordwestlichen Maindreieck vom Neolithikum bis zum Ende des Mittelalters. Würzburger Arbeiten zur Prähistorischen Archäologie 4. Rahden/Westfalen: Leidorf; 2012.

[56] Balfanz I. Die ur- und frühgeschichtliche Besiedlung des Kreises Riesa-Großenhain (Reg.-Bez. Dresden). Halle (Saale): Martin-Luther-Universität Halle-Wittenberg; 2003. 10.25673/2770

[57] Wegener R. Studien zum vorgeschichtlichen Siedlungswesen in Nordwestsachsen. Halle (Saale): Universitäts- und Landesbibliothek Sachsen-Anhalt; 2014. 10.25673/1481

[58] Schülke A. Landschaften–eine archäologische Untersuchung der Region zwischen Schweriner See und Stepenitz. Römisch-germanische Forschungen 68. Darmstadt: Zabern; 2011.

[59] Saile T. Untersuchungen zur ur- und frühgeschichtlichen Besiedlung der nördlichen Wetterau, Teil 1: Text. Materialien zur Vor- und Frühgeschichte von Hessen 21. Wiesbaden: Landesamt für Denkmalpflege Hessen; 1998.

[60] European Environment Agency. EEA Catchments and Rivers Network System ECRINS v1.1. Rationales, building and improving for widening uses to Water Accounts and WISE applications. EEA Technical report 7/2012. Copenhagen: European Environment Agency; 2012. 10.2800/51667

[61] Jarvis A, Reuter HI, Nelson A, Guevara E. Hole-filled seamless SRTM data V4, International Centre for Tropical Agriculture (CIAT). 2008 [Cited 2021 November 24]. Available from: http://srtm.csi.cgiar.org

[62] Farr TG, Rosen PA, Caro E, Crippen R, Duren R, Hensley S, et al. The Shuttle Radar Topography Mission. Rev Geophys. 2007; 45(2): 1–33. 10.1029/2005RG000183

[63] Reuter H, Nelson A, Jarvis A. An Evaluation of Void-Filling Interpolation Methods for SRTM Data. Int J Geogr Inf Sci. 2007; 21(9):983–1008. 10.1080/13658810601169899

[64] Ballasus H, Schneider B, Suchodoletz H von, Miera J, Werban U, Fütterer P, et al. Overbank silt-clay deposition and intensive Neolithic land use in a Central European catchment–Coupled or decoupled? Sci Total Environ. 2022; 806:150858, doi: 10.1016/j.scitotenv.2021.150858 34627920

[65] Suchodoletz H von, Pohle M, Khosravichenar A, Ulrich M, Hein M, Tinapp C, et al. The fluvial architecture of buried floodplain sediments of the Weiße Elster River (Germany) revealed by a novel method combination of drill cores with two-dimensional and spatially resolved geophysical measurements. Earth Surf Process Landf., 2022;1–22. 10.1002/esp.5296

[66] Ssymank A. Neue Anforderungen im europäischen Naturschutz. Das Schutzgebietssystem Natura 2000 und die „FFH-Richtlinie der EU“. Natur und Landschaft. 1994; 69:395–406.

[67] Potschin M, Bastian O. Landscapes and landscape research in Germany. Belgeo. 2004; 2–3:265–276. 10.4000/belgeo.13688

[68] Schwarzer M, Mengel A, Konold W, Reppin N, Mertelmeyer L, Jansen M, et al. Bedeutsame Landschaften in Deutschland–Gutachtliche Empfehlungen für eine Raumauswahl, Band 1: Schleswig-Holstein und Hamburg, Niedersachsen und Bremen, Mecklenburg-Vorpommern, Nordrhein-Westfalen, Sachsen-Anhalt, Brandenburg und Berlin. Bfn-Skripten 516. Bonn: Bundesamt für Naturschutz; 2018.

[69] Schwarzer M, Mengel A, Konold W, Reppin N, Mertelmeyer L, Jansen M, et al. Bedeutsame Landschaften in Deutschland–Gutachtliche Empfehlungen für eine Raumauswahl, Band 2: Rheinland-Pfalz, Saarland, Hessen, Thüringen, Sachsen, Baden-Württemberg, Bayern. Bfn-Skripten 517. Bonn: Bundesamt für Naturschutz; 2018.

[70] Emmert U, Horsting VG, Stettner G, Zitzmann A. Geologische Übersichtskarte der Bundesrepublik Deutschland 1:200.000, Blatt CC 6334 Bayreuth. Hannover: Bundesanstalt für Geowissenschaften und Rohstoffe; 1981.

[71] Kriebel U, Martiklos G, Standke G, Knoth W. Geologische Übersichtskarte der Bundesrepublik Deutschland 1:200.000, Blatt CC 4734 Leipzig. Hannover: Bundesanstalt für Geowissenschaften und Rohstoffe; 1998.

[72] Radzinski KH, Kästner H, Seidel G, Wiefel H, Berger HJ, Zitzmann A. Geologische Übersichtskarte der Bundesrepublik Deutschland 1:200.000, Blatt CC 5534 Zwickau. Hannover: Bundesanstalt für Geowissenschaften und Rohstoffe; 1999.

[73] Stremme H. Bodenkarte der Deutschen Demokratischen Republik. Bodenkunde und Bodenkultur 1. Leipzig: Bibliographisches Institut; 1951.

[74] Kasch W, Jacke W, Knott K. 3 Karten der Deutschen Demokratischen Republik: Bodengüte, Bearbeitungsschwere, Kalkgehalt. Bodenkunde und Bodenkultur 2. Leipzig: Bibliographisches Institut; 1953.

[75] Kasch W, Sahle E, Lorenz P. Bodentypen mit farbigen Bodenprofilen aus der Deutschen Demokratischen Republik. Bodenkunde und Bodenkultur 3. Leipzig: Bibliographisches Institut; 1954.

[76] DWD Climate Data Center (CDC). Multi-annual means of grids of air temperature (2m) over Germany 1981–2010, version v1.0. 2018 [Cited 2021 November 24]. Available from: ftp://opendata.dwd.de/climate_environment/CDC/grids_germany/multi_annual/

[77] DWD Climate Data Center (CDC). Multi-annual grids of precipitation height over Germany 1981–2010, version v1.0. 2018 [Cited 2021 November 24]. Available from: ftp://opendata.dwd.de/climate_environment/CDC/grids_germany/multi_annual/

[78] DWD Climate Data Center (CDC). Multi-annual grids of annual sunshine duration over Germany 1981–2010, version v1.0. 2018 [Cited 2021 November 24]. Available from: ftp://opendata.dwd.de/climate_environment/CDC/grids_germany/multi_annual/

[79] DWD Climate Data Center (CDC). Multi-annual grids of number of frost days over Germany, version v1.0. 2018 [Cited 2021 November 24]. Available from: ftp://opendata.dwd.de/climate_environment/CDC/grids_germany/multi_annual/

[80] Hartwich R, Behrens J, Eckelmann W, Haase G, Richter A, Roeschmann G, et al. Bodenübersichtskarte der Bundesrepublik Deutschland im Maßstab 1: 1 000 000 (BÜK 100). Hannover: Bundesanstalt für Geowissenschaften und Rohstoffe; 1998.

[81] Panagos P, Van Liedekerke M, Jones A, Montanarella L. “European Soil Data Centre: Response to European policy support and public data requirements”. Land Use Policy. 2012; 29:329–338. doi: 10.1016/j.landusepol.2011.07.003

[82] Vogt JV, Soille P, de Jager A, Rimaviciute E, Mehl W, Foisneau S, et al. A pan-European River and Catchment Database. Luxembourg: Office for Official Publications of the EC; 2007.

[83] European Environment Agency. CLC2006 technical guidelines. EEA Technical report 17/2007. Luxembourg: Office for Official Publications of the European Communities; 2007. 10.2800/12134

[84] Conrad O, Bechtel B, Bock M, Dietrich H, Fischer E, Gerlitz L, et al. System for Automated Geoscientific Analyses (SAGA) v. 2.1.4. Geosci Model Dev. 2015; 8:1991–2007. 10.5194/gmd-8-1991-2015

[85] QGIS Development Team. QGIS Geographic Information System. Open Source Geospatial Foundation Project. 2021 [Cited 2021 November 24]. Available from: http://qgis.osgeo.org doi: 10.1071/AH19252 33211999

[86] Eggert MKH. Prähistorische Archäologie: Konzepte und Methoden. UTB 2092, fourth ed. Tübingen: Francke; 2012.

[87] Eggert MKH, Samida S. Ur- und frühgeschichtliche Archäologie. UTB 3254. Tübingen: Francke; 2013.

[88] Fischer U. Über Nachbestattungen im Neolithikum von Sachsen-Thüringen. In: Klumbach H., editor. Festschrift des Römisch-Germanisches Zentralmuseums in Mainz zur Feier seines hundertjährigen Bestehens 1952, volume 3. Mainz: Römisch-Germanisches Zentralmuseum; 1953. pp. 161–181.

[89] Mildenberger G. Die großen Grabhügel der mitteldeutschen Jungsteinzeit: ein Beitrag zur Chronologie und Kulturgeschichte des Neolithikums. Veröffentlichungen des Landesmuseums für Vorgeschichte Dresden 2. Leipzig: Bibliographisches Institut; 1953.

[90] Behrens H. Die Jungsteinzeit im Mittelelbe-Saale-Gebiet. Veröffentlichungen des Landesamtes für Denkmalpflege und Archäologie Sachsen-Anhalt 27. Berlin: Deutscher Verlag der Wissenschaften; 1973.

[91] Kaufmann D. Produktivkräfte und Kulturwandel im Neolithikum. In: Horst F, editor. Produktivkräfte und Produktionsverhältnisse in ur- und frühgeschichtlicher Zeit. Historiker-Gesellschaft der DDR: Tagung der Fachgruppe Ur- und Frühgeschichte 11. Berlin: Akademie-Verlag; 1985. pp. 31–40.

[92] Beran J. Untersuchungen zur Stellung der Salzmünder Kultur im Jungneolithikum des Saalegebietes. Beiträge zur Ur- und Frühgeschichte Mitteleuropas 2. Wilkau-Haßlau: Beier und Beran; 1993.

[93] Beier HJ. Die Kulturengliederung im jüngeren Mittelneolithikum des Mittelelbe-Saale-Gebietes. Ausgrab Funde. 1993; 38:173–178.

[94] Lüning J. Erneute Gedanken zur Benennung der neolithischen Perioden. Germania. 1996; 74:233–237.

[95] Preuss J. Das Neolithikum in Mitteleuropa 1,1 Teil A. Wilkau-Haßlau: Beier und Beran; 1998.

[96] Müller J. Soziochronologische Studien zum Jung- und Spätneolithikum im Mittelelbe-Saale-Gebiet (4100–2700 v. Chr.). Eine sozialhistorische Interpretation prähistorischer Quellen. Vorgeschichtliche Forschungen 21. Rahden/Westfalen: Leidorf; 2001.

[97] Driehaus J. Die Altheimer Gruppe und das Jungneolithikum in Mitteleuropa. Mainz: Römisch-Germanisches Zentralmuseum; 1960.

[98] Becker CJ. Die mittel-neolithischen Kulturen in Südskandinavien. Acta Archaeologica. 1955; 25:49–150.

[99] Ostritz S. Untersuchungen zur Siedlungsplatzwahl im mitteldeutschen Neolithikum. Beiträge zur Ur- und Frühgeschichte Mitteleuropas 25. Weissbach: Beier und Beran; 2000.

[100] Hinz M. Neolithische Siedlungsstrukturen im südöstlichen Schleswig-Holstein: Dynamik in Landschaft und Besiedlung. Frühe Monumentalität und soziale Differenzierung 3. Bonn: Habelt; 2014.

[101] Hilbig O. Zur Besiedlungsgeschichte im Gebiet um den Göttwitzer See, Kr. Grimma (Sachsen). Arbeits- und Forschungsberichte zur sächsischen Bodendenkmalpflege. 1993; 36:7–65.

[102] Pankau C. Methodische Fragen bei der zeitlichen Klassifizierung und räumlichen Fixierung von Fundstellen im Rahmen von Besiedlungsstudien. Ein Fallbeispiel von der östlichen Schwäbischen Alb. Archäologische Informationen. 2004; 27:245–249. 10.11588/ai.2004.2

[103] Stäuble H, Meller H, Tinapp C. Die Tagebaue im Südraum Leipzig. In: Geupel V, editor. Leipzig und sein Umland: Archäologie zwischen Elster und Mulde. Führer zu archäologischen Denkmälern in Deutschland 32. Stuttgart: Theiss; 1996. pp. 223–241. doi: 10.1016/0014-5793(96)00605-9

[104] Stäuble H. Braunkohlen- und Trassenarchäologie: eine Herausforderung mit Tradition. Arbeits- und Forschungsberichte zur sächsischen Bodendenkmalpflege, Beiheft. 2010; 21:67–82.

[105] Gerlach R. Holozän: Die Umgestaltung der Landschaft durch den Menschen seit dem Neolithikum. Jahrbuch des Rheinischen Vereins für Denkmalpflege und Landschaftsschutz. 2006; 2005:87–98.

[106] Gerlach R. Meurers-Balke J. Trockentäler im Neolithikum–neue Bäche in der Eisenzeit. Geogr Rundsch. 2017; 69:32–33.

[107] Schiffer MB. Formation Processes of the Archaeological Record. Albuquerque: University of Mexico Press; 1987.

[108] Gerhard S. Beiträge zur archäologischen Quellenkritik an Beispielen aus dem Neolithikum und der Frühbronzezeit Südbayerns. Arbeiten zur Archäologie Süddeutschlands 18. Büchenbach: Faustus; 2006.

[109] Miera JJ. Ursachen, Formen und Konsequenzen des Erzählens in der Prähistorischen Archäologie: eine Synthese der deutschsprachigen Theoriedebatte. Forum Kritische Archäologie. 2019; 8:1–24. 10.6105/journal.fka.2019.8.1

[110] Wilbertz OH. Die Urnenfelderkultur in Unterfranken. Materialhefte zur bayerischen Vorgeschichte. Reihe A–Fundinventare und Ausgrabungsbefunde 49. Kallmünz/Opf: Lassleben; 1982.

[111] Zimmermann A, Wendt KP. Wie viele Bandkeramiker lebten 5.060 v. Chr.? Techniken Geographischer Informationssysteme zum Schätzen von Bevölkerungsdichten. Archäologische Informationen 2003; 26:491–497. 10.11588/ai.2003.2.12712

[112] Frank T. Zur Bedeutung der Tätigkeit von Sammlern für die Archäologie. Die Kunde, Neue Folge. 2007; 58:91–106.

[113] Ahlrichs JJ, Henkner J, Schmidt K. A seamless workflow for defining archaeological site densities with contour lines by using the open source (geo-)statistical language R. Collaborative Research Center 1070 –Geoscientific and archaeological research. Technical note. 2016; 1. [Cited 2021 November 24]. Available from: https://uni-tuebingen.de/de/75294

[114] Saile T. Reliefenergie als innere Gültigkeitsgrenze der Fundkarte. Germania. 2001; 79:93–120.

[115] Saile T. Relief Intensity and the Formation of the Archaeological Record. Prehistoria. 2001; 1:91–101.

[116] Breiman L. Random Forests. Machine Learning. 2001; 45:5–32. 10.1023/A:1010933404324

[117] Cutler A, Cutler RD, Stevens JR. Random Forests. In: Zhang C, Ma Y, editors. Ensemble Machine Learning: Methods and Applications. Boston: Springer; 2012. pp. 157–175. 10.1007/978-1-4419-9326-7

[118] Janitza S, Hornung R. On the overestimation of random forest’s out-of-bag error. PLoS ONE. 2018; 13:e0201904. doi: 10.1371/journal.pone.0201904 30080866 PMC6078316

[119] Stehman S. Selecting and interpreting measures of thematic classification accuracy. Remote Sens Environ. 1997; 62:77–89. 10.1016/S0034-4257(97)00083-7

[120] van Rijsbergen CJ. Information retrieval. Second ed. London: Butterworths; 1979.

[121] Cohen J. A coefficient of agreement for nominal scales. Educ Psychol Meas. 1960; 20:37–46. 10.1177/001316446002000104

[122] Fleiss JL. Measuring nominal scale agreement among many raters. Psychol Bull. 1971; 76:378–382. 10.1037/h0031619

[123] Landis JR, Koch GG. The measurement of observer agreement for categorical data. Biometrics. 1977; 33:159–174. 10.2307/2529310 843571

[124] McHugh ML. Interrater Reliability: The Kappa Statistic. Biochem Med (Zagreb). 2012; 22:276–282. PMCID: PMC3900052 23092060 PMC3900052

[125] Congalton RG, Green K. Assessing the accuracy of remotely sensed data: principles and practices. Boca Raton: CRC press; 2008. 10.1201/9781420055139

[126] Hounkpatin KOL, Schmidt K, Stumpf F, Forkuor G, Behrens T, Scholten T, et al. Predicting reference soil groups using legacy data: A data pruning and Random Forest approach for tropical environment (Dano catchment, Burkina Faso). Scientific Reports. 2018; 8:9959. doi: 10.1038/s41598-018-28244-w 29967391 PMC6028482

[127] Raghavan V, Bollmann P, Jung GS. A critical investigation of recall and precision as measures of retrieval system performance. ACM Trans Inf Syst. 1989; 7:205–229. 10.1145/65943.65945

[128] Strobl C, Boulesteix AL, Kneib T, Augustin T, Zeileis A. Conditional variable importance for random forests. BMC Bioinformatics. 2008; 9:307. doi: 10.1186/1471-2105-9-307 18620558 PMC2491635

[129] Genuer R, Poggi JM, Tuleau-Malot C. Variable selection using Random Forests. Pattern Recognit Lett. 2010; 31:2225–2236. 10.1016/j.patrec.2010.03.014

[130] Dormann CF, Elith J, Bacher S, Buchmann C, Carl G, Carré G, et al. Collinearity: a review of methods to deal with it and a simulation study evaluating their performance. Ecography. 2013; 36:027–046. 10.1111/j.1600-0587.2012.07348.x

[131] Liaw A, Wiener M. 2002. Classification and Regression by randomForest. R News. 2002; 2(3):8–22.

[132] Kuhn M. Building Predictive Models in R Using the caret Package. J Stat Softw. 2008; 28(5):1–26. 10.18637/jss.v028.i0527774042

[133] Kuhn M. Caret: classification and regression training. Astrophysics Source Code Library, 2005; 1:5003. Bibcode: 2015ascl.soft05003K

[134] R Core Team. A language and environment for statistical computing. R Foundation for Statistical Computing, Vienna. 2020 [Cited 2021 November 24]. Available from: https://www.R-project.org/

[135] Koschik H. Die Bronzezeit im südwestlichen Oberbayern. Materialhefte zur bayerischen Vorgeschichte. Reihe A–Fundinventare und Ausgrabungsbefunde 50. Kallmünz/Opf: Lassleben; 1981.

[136] Vogt R, Kretschmer I. Archaeology and agriculture: conflicts and solutions. E & G Quaternary Science Journal. 2019; 68:47–51. 10.5194/egqsj-68-47-2019

[137] Kempf M. Modeling multivariate landscape affordances and functional ecosystem connectivity in landscape archeology. Archaeol Anthropol Sci. 2020; 12:159. 10.1007/s12520-020-01127-w

[138] Ihm P, Lüning J, Zimmermann A. Statistik in der Archaeologie: Probleme der Anwendung, allgemeine Methoden, Seriation und Klassifikation. Archaeo-Physika 9. Bonn: Habelt; 1978. doi: 10.1016/0005-2760(78)90113-3

[139] Shennan S. Quantifying Archaeology. Edinburgh: Edinburgh University Press; 1988.

[140] Barceló JA. Chi‐Square Analysis. In: López Varela SL, editor. The Encyclopedia of Archaeological Sciences. Malden: Wiley-Blackwell; 2018. 10.1002/9781119188230.saseas0090

[141] Ahlrichs JJ, Henkner J, Schmidt K, Scholten T, Kühn P, Knopf T. Bronzezeitliche Siedlungsdynamiken zwischen der Baar und angrenzenden Naturräumen. In: Neumann D, Nessel B, Bartelheim M, editors. Bronzezeitlicher Transport: Akteure, Mittel und Wege. RessourcenKulturen 8. Tübingen: Tübingen University Press; 2018. pp. 269–303. 10.15496/publikation-26722

[142] Zimmermann A, Richter J, Frank T, Wendt KP. Landschaftsarchäologie II–Überlegungen zu Prinzipien einer Landschaftsarchäologie, Bericht der Römisch-Germanischen Kommission. 2005; 85:37–95.

[143] Zimmermann A, Hilpert J, Wendt KP. Estimations of Population Density for Selected Periods Between the Neolithic and AD 1800. Hum Biol. 2009; 81:357–380. doi: 10.3378/027.081.0313 19943751

[144] Higgs ES. Papers in Economic Prehistory. Studies by Members and Associates of the British Academy Major Research Project in the Early History of Agriculture. Cambridge: Cambridge University press; 1972.

[145] Higgs ES. Palaeoeconomy: being the second volume of Papers in Economic Prehistory by members and associates of the British Academy Major Research Project in the Early History of Agriculture. Cambridge: Cambridge University press; 1975.

[146] Jarman MR, Bailey GN, Jarman HN. Early european Agriculture. Its foundation and development: being the third volume of Papers in Economic Prehistory by members and associates of the British Academy Major Research Project in the Early History of Agriculture. Cambridge: Cambridge University press; 1982.

[147] Vita-Finzi C, Higgs ES. Prehistoric Economy in the Mount Carmel Area of Palestine: Site Catchment Analysis. With Contributions by D. Sturdy–J. Harriss–A. J. Legge–H. Tippett. Proceedings of the Prehistoric Society. 1970; 36:1–37. 10.1017/S0079497X00013074

[148] Higgs ES, Vita-Finzi C. Prehistoric economies: a territorial approach. In: Higgs ES, editor. Papers in Economic Prehistory. Studies by Members and Associates of the British Academy Major Research Project in the Early History of Agriculture. Cambridge: Cambridge University press, Cambridge; 1972. pp. 27–38.

[149] Jarman MR. A territorial model for archaeology: a behavioral and geographical approach. In: Clarke DL, editor. Models in Archaeology. London: Methuen; 1972. pp. 705–733.

[150] Becker D, De Andrés-Herrero M, Willmess C, Weniger GC, Bareth G. Investigating the Influence of Different DEMs on GIS-Based Cost Distance Modeling for Site Catchment Analysis of Prehistoric Sites in Andalusia. ISPRS Int J Geoinf. 2017; 6:36. 10.3390/ijgi6020036

[151] Schmidt I, Hilpert J, Kretschmer I, Peters R, Broich M, Schiesberg S, et al. Approaching prehistoric demography: proxies, scales and scope of the Cologne Protocol in European contexts. Philos Trans R Soc Lond B Biol Sci. 2020; 376:20190714. doi: 10.1098/rstb.2019.0714 33250025 PMC7741091

[152] Jarman MR, Vita-Finzi C, Higgs ES. Site catchment analysis in archaeology. In: Ucko PJ, editor. Man, settlement and urbanism: proceedings of a meeting of the research seminar in archaeology and related subjects held at the institute of archaelogy. Gloucester Crescent: Duckworth; 1972. pp. 61–66.

[153] Jarman MR. Prehistoric economic development in sub-Alpine Italy. In: Sieveking G de G, Longworth IH, Wilson KE, editors. Problems in Economic and Social Archaeology. London: Duckworth; 1976. pp. 523–548.

[154] Kreuz AM. Die ersten Bauern Mitteleuropas. Eine archäobotanische Untersuchung zu Umwelt und Landwirtschaft der ältesten Bandkeramik. Analecta praehistorica Leidensia 23. Leiden: University of Leiden; 1990.

[155] Posluschny AG. Over the Hills and Far Away? Cost Surface Based Models of Prehistoric Settlement Hinterlands. In: Frischer B, Webb Crawford J, Koller D, editors. Making History Interactive. Computer Applications and Quantitative Methods in Archaeology (CAA). Proceedings of the 37th International Conference, Williamsburg/VA, United States of America, March 22–26, 2009. BAR International Series S2079. Oxford: Archaeopress; 2010. pp. 313–319.

[156] Posluschny AG, Fischer E, Rösch M, Schatz K, Stephan E, Stobbe A. Modelling the Agricultural Potential of Early Iron Age Settlement Hinterland Areas in Southern Germany. In: Kluiving SJ, Guttmann-Bond EB, editors. Landscape Archaeology between Art and Science. From a Multi to an Interdisciplinary Approach. Amsterdam: Amsterdam University Press; 2012. pp. 413–428.

[157] Herbig C, Maier U, Elburg R, Stäuble H. „Neolithische Füllhörner“. Archäobotanische Untersuchungen in fünf linienbandkeramischen Brunnen in Westsachsen. Offa. 2013; 69/70:265–293.

[158] Kretschmer S, Viol P, Herbig C, Muigg B, Tegel W, Tinapp C. Der Fundplatz Droßdorf im Tagebaufeld Peres (Lkr. Leipzig). Ein früh-, mittel- und spätneolithisches Siedlungsareal mit zahlreichen Brunnen. Ausgrabungen in Sachsen. 2016; 5:30–57.

[159] Schell F, Herbig C. Ein linienbandkeramischer Brunnen im Labor: Die Ausgrabung einer Blockbergung (Bef. 3682) aus Droßdorf (Lkr. Leipzig). Ausgrabungen in Sachsen. 2018; 6:16–36.

[160] Ahlrichs JJ, Gries P, Schmidt K. Distance relationships or does distance matter–a non-isotropic spatial relationship by integrating human energy expenditure in terrain based estimations–Seamless workflow for defining archaeological Site Exploitation Territories (SET) by using the open source (geo-)statistical language R. Collaborative Research Center 1070 –Geoscientific and archaeological research. Technical note. 2016; 3. [Cited 2021 November 24]. Available from: https://uni-tuebingen.de/en/91679

[161] Tobler WR. Three presentations on geographical analysis and modelling: non-isotrophic modelling, speculations on the geometry of geography, global spatial analysis. National Center for Geographic Information and Analysis. Technical Report 93–1. 1993 [Cited 2021 November 24]. Available from: https://escholarship.org/uc/item/05r820mz

[162] Gorenflo LJ, Gale N. Mapping Regional Settlement in Information Space. J Anthropol Archaeol. 1990; 9:240–274. 10.1016/0278-4165(90)90008-2

[163] Herzog I. A review of case studies in archaeological least-cost analysis. Archaeologia e Calcolatori. 2014; 25:223–239.

[164] Imhof E. Gelände und Karte. Zürich: Rentsch; 1950.

[165] Baumann W, Fritzsche C, Coblenz W. Stratigraphische Befunde zur Schnurkeramik in einem Grabhügel bei Werben, Kr. Leipzig. Ausgrab Funde. 1983; 28:1–5.

[166] Sabel KJ. Die Bedeutung der physisch-geographischen Raumausstattung für das Siedlungsverhalten der frühesten Bandkeramik in der Wetterau (Hessen). Praehistorische Zeitschrift. 1983; 58:158–172. 10.1515/prhz.1983.58.2.158

[167] Eckmeier E, Pätzold S, Lehndorff E, Gerlach R. Geochemische Untersuchungen von Böden zur Rekonstruktion der prähistorischen Landnutzungsgeschichte. In: Bork HR, Meller H, Gerlach R, editors. Umweltarchäologie-Naturkastrophen und Umweltwandel im archäologischen Befund. 3. Mitteldeutscher Archäologentag vom 7.-9- Oktober 2010. Tagungen Des Landesmuseums für Vorgeschichte Halle (Saale) 6. Halle (Saale): Landesamt für Denkmalpflege und Archäologie Sachsen-Anhalt; 2011. pp. 37–45.

[168] Ballabio C, Panagos P, Lugato E, Huang JH, Orgiazzi A, Jones A, et al. Copper distribution in European topsoils: An assessment based on LUCAS soil survey. Sci Total Environ. 2018; 636:282–298. doi: 10.1016/j.scitotenv.2018.04.268 29709848

[169] Panagos P, Ballabio C, Lugato E, Jones A, Borrelli P, Scarpa S, et al. Potential Sources of Anthropogenic Copper Inputs to European Agricultural Soils. Sustainability. 2018; 10:2380. 10.3390/su10072380

[170] Billig G. Ur- und Frühgeschichte des sächsischen Vogtlandes eine populärwissenschaftliche Einführung in die urgeschichtliche Heimatforschung und ein Führer zur ur- und frühgeschichtlichen Abteilung des vogtländischen Kreismuseums in Plauen. Vogtländisches Kreismuseum Plauen, Museumsreihe 5. Plauen: Rat des Stadtkreises Plauen; 1954. doi: 10.1007/BF01567066

[171] Simon K. Beiträge zur Urgeschichte des Vogtlandes I: Archäologische Quellen. Arbeits- und Forschungsberichte zur sächsischen Bodendenkmalpflege. 1989; 33:11–226.

[172] Simon K. Beiträge zur Urgeschichte des Vogtlandes II. Kulturgeschichtliche Auswertung. Arbeits- und Forschungsberichte zur sächsischen Bodendenkmalpflege. 1991; 34:63–156.

[173] Valde-Nowak P. Siedlungsarchäologische Untersuchungen zur neolithischen Nutzung der mitteleuropäischen Gebirgslandschaften. Internationale Archäologie 69. Rahden/Westfalen: Leidorf; 2002.

[174] Valde-Nowak P, Kienlin TL. Neolithische Transhumanz in den Mittelgebirgen: Ein Survey im westlichen Schwarzwald. Praehistorische Zeitschrift. 2002; 77:29–75. 10.1515/prhz.2002.77.1.29

[175] Kienlin TL, Valde-Nowak P. Neolithic Transhumance in the Black Forest Mountains, SW Germany. J Field Archaeol. 2004; 29:29–44. 10.1179/jfa.2004.29.1-2.29

[176] Tinapp C, Meller H, Baumhauer R. Holocene accumulation of colluvial and alluvial sediments in the weiße elster river valley in saxony, Germany. Archaeometry. 2008; 50:696–709. 10.1111/j.1475-4754.2007.00356.x

[177] Tinapp C, Stäuble H. Topographie, Geologie, Boden und moderne Nutzung der Grabungsflächen. In: Stäuble H, Veit U, editors. Der bandkeramische Siedlungsplatz Eythra in Sachsen: Studien zur Chronologie und Siedlungsentwicklung. Leipziger Forschungen zur ur- und frühgeschichtlichen Archäologie 9. Leipzig: Professur für Ur- und Frühgeschichte der Universität Leipzig; 2016. pp. 19–26.

[178] Tinapp C., Heine Y., Heinrich S, Herbig C, Schneider B, Stäuble H, et al. Die Pleißeaue südlich von Leipzig. Geoarchäologische Erkenntnisse zur stratigraphischen Position archäologischer Fundstellen im unteren Pleißetal. Ausgrabungen in Sachsen. 2020; 7:7–19.

[179] Tinapp C, Stäuble H. Auenentwicklung und Besiedlungsgeschichte im Tal der Weissen Elster südlich von Leipzig, Trierer Geographische Studien. 2000; 23:31–48.

[180] Tinapp C. Geoarchäologische Untersuchungen zur holozänen Landschaftsentwicklung der südlichen Leipziger Tieflandsbucht. Trierer Geographische Studien 26. Trier: Geographische Gesellschaft; 2002.

[181] Brown AG. Alluvial geoarchaeology: Floodplain archaeology and environmental change. Cambridge: Cambridge University Press; 1997.

[182] Bard KA. An introduction to the archaeology of Ancient Egypt. Second Edn. Chichester: Wiley Blackwell; 2015.

[183] Suchodoletz H von, Richter C, Walther F, Bliedtner M, Eloshvili M, Losaberidze L, et al. Snail assemblages in Holocene floodplain research–an example from the southern Caucasus. E & G Quaternary Science Journal. 2020; 69:247–260. 10.5194/egqsj-69-247-2020

[184] Kubenz T. Baalberger Kultur. In: Beier HJ, Einicke R, editors. Das Neolithikum im Mittelelbe-Saale-Gebiet und in der Altmark: Eine Übersicht und ein Abriß zum Stand der Forschung. Beiträge zur Ur- und Frühgeschichte Mitteleuropas 4. Wilkau-Haßlau: Beier und Beran; 1994. pp. 113–128.

[185] Montag T. Kugelamphorenkultur (KAK). In: Beier HJ, Einicke R., editors. Das Neolithikum im Mittelelbe-Saale-Gebiet und in der Altmark: Eine Übersicht und ein Abriß zum Stand der Forschung. Beiträge zur Ur- und Frühgeschichte Mitteleuropas 4. Wilkau-Haßlau: Beier und Beran; 1994. pp. 215–228.

[186] Schindler G. Salzmünder Kultur. In: Beier HJ, Einicke R, editors. Das Neolithikum im Mittelelbe-Saale-Gebiet und in der Altmark: Eine Übersicht und ein Abriß zum Stand der Forschung. Beiträge zur Ur- und Frühgeschichte Mitteleuropas 4. Wilkau-Haßlau: Beier und Beran; 1994. pp. 145–158.

[187] Heynowski R, Reiß R., editors. Atlas zur Geschichte und Landeskunde von Sachsen. BI 1.1–1.5. Ur- und Frühgeschichte Sachsens: Beiheft zur Karte B I 1.1–1.5. Leipzig: Sächsische Akademie der Wissenschaften zu Leipzig; 2010.

[188] Schoknecht U. Typentafeln zur Ur- und Frühgeschichte, 1. Ergänzung. Weimar: Kulturbund der Deutschen Demokratischen Republik; 1980.

[189] Neolithikum Raetzel-Fabian D. Göttinger Typentafeln zur Ur- und Frühgeschichte Mitteleuropas. Göttingen: Arbeitsgruppe Typentafeln; 1983.

[190] Fischer U. Mitteldeutschland und die Schnurkeramik. Jahresschrift für Mitteldeutsche Vorgeschichte. 1958; 41/42:254–298.

[191] Torbrügge W. Die Bronzezeit in der Oberpfalz. Materialhefte zur bayerischen Vorgeschichte 13. Kallmünz/Opf: Lassleben; 1959.

[192] Torbrügge W. Die Hallstattzeit in der Oberpfalz I: Auswertung und Gesamtkatalog, Text. Materialhefte zur bayerischen Vorgeschichte. Reihe A–Fundinventare und Ausgrabungsbefunde 39. Kallmünz/Opf: Lassleben; 1979.

[193] Friederich S, Kleinecke J. Die schnurkeramische Siedlung Profen, Burgenlandkreis. In: Meller H, Friederich S, Küßner M, Stäuble H, Risch R, editors. Siedlungsarchäologie des Endneolithikums und der frühen Bronzezeit, 11. Mitteldeutscher Archäologentag vom 18. bis 20. Oktober 2018 in Halle (Saale). Tagungen des Landesmuseums für Vorgeschichte Halle 20/1. Halle (Saale): Landesamt für Denkmalpflege und Archäologie Sachsen-Anhalt; 2019. pp. 243–252.

[194] Kretzsch H. Drei schnurkeramische Grabanlagen auf dem „Großem Steine”bei Seifartsdorf, Landkreis Eisenberg/Thür. Alt Thuring. 1955; 1:182–209.

[195] Höckner H. Ausgrabung von schnurkeramischen Grabhügeln und Siedelplätzen im Luckaer Forst, Kreis Altenburg. Arbeits- und Forschungsberichte zur sächsischen Bodendenkmalpflege. 1957; 6:58–181.

[196] Herklotz L. Die Ausgrabung schnurkeramischer Hügelgräber und Siedlungsplätze im Luckaer Forst, Kr. Altenburg. In: Vogt HJ, editor. Archäologische Feldforschungen in Sachsen: Fünfzig Jahre Landesmuseum für Vorgeschichte Dresden. Arbeits- und Forschungsberichte zur Sächsischen Bodendenkmalpflege, Beiheft 18. Berlin: Deutscher Verlag der Wissenschaften; 1988. pp. 79–81.

[197] Kretschmer S. Baalbergezeitliche Siedlungsspuren mit Brunnen und einer Trapezgrabenanlage vom Fundplatz Droßdorf (Lkr. Leipzig): Ein Vorbericht. In: Bertemes F, Rück O, editors. Alteuropäische Forschungen Neue Folge 9: Neue Forschungen und Aspekte zur Baalberger Kultur. Langenweissbach: Beier und Beran; 2016. pp. 147–156.

[198] Bartelt U. Beste Wohnlage am Auenrand der Weißen Elster–Siedlungsbefunde vom Frühneolithikum bis in die Eisenzeit bei Großdalzig, Lkr. Leipziger Land. Arbeits- und Forschungsberichte zur sächsischen Bodendenkmalpflege. 2004; 46:115–173.

[199] Bergemann S. Zauschwitz (Landkreis Leipzig): Siedlungen und Gräber eines neolithischen Fundplatzes. Universitätsforschungen zur Prähistorischen Archäologie 314. Bonn: Habelt; 2018.

[200] Walter D, Bücke S, Schulze J. Beiträge zur Archäologie der Erfurter Mulde I. Alt Thuring. 1987; 22/23:63–164.

[201] Kaufmann D. Die jungsteinzeitliche Besiedlung am unteren Bodelauf unter Berücksichtigung siedlungskundlicher Probleme. Jahresschrift für mitteldeutsche Vorgeschichte. 1967; 60:89–110.

[202] Starling NJ. Neolithic settlement patterns in Central Germany. Oxford Journal of Archaeology. 1983; 2:1–11. 10.1111/j.1468-0092.1983.tb00092.x

[203] Christl A. Verschiebungen der Höhengrenzen der ur- und frühgeschichtlichen Besiedlung am Erzgebirge. Alteuropäische Forschungen 5. Langenweissbach: Beier und Beran; 2004.

[204] Jacob H. Die ur- und frühgeschichtliche Besiedlung zwischen Dresdener Elbtalweitung und Oberem Osterzgebirge. Arbeits- und Forschungsberichte zur sächsischen Bodendenkmalpflege. 1982; 24/25:25–137.

[205] Heinrich W, Lange E. Ein Beitrag zur Kenntnis der Waldgeschichte des Thüringisch-Sächsischen Vogtlandes. Feddes Repert. 1969; 80:437–462. 10.1002/fedr.19690800411

[206] Mildenberger G. Pollenanalyse und Siedlungsgeschichte im Vogtland. Praehistorische Zeitschrift. 1972; 47:110–115. 10.1515/prhz.1972.47.1-2.105

[207] Jacob H. Pollenanalytische Untersuchungen der Torfschichten des Göttwitzer Sees bei Wermsdorf, Bezirk Leipzig. Arbeits- und Forschungsberichte zur sächsischen Bodendenkmalpflege. 1957; 6:117–330.

[208] Jacob H. Pollenanalysen aus dem Gebiet des ehemaligen Göttwitzer Sees bei Mutzschen, Kr. Grimma. Arbeits- und Forschungsberichte zur sächsischen Bodendenkmalpflege. 1971; 19:159–175.

[209] Schlette F. Beziehungen zwischen Mensch und natürlicher Umwelt im nördlichen und östlichen Harzvorland. In: Schlette F, editor. Urgeschichtliche Besiedlung in ihrer Beziehung zur natürlichen Umwelt. Wissenschaftliche Beiträge der Martin-Luther-Universität Halle-Witternberg 1980/6 (L 15). Halle (Saale): Abteilung Wissenschaftspublizistik der Martin-Luther-Universität Halle-Wittenberg; 1980. pp. 41–56.

[210] Schwarz K. Lagen die Siedlungen der linearbandkeramischen Kultur Mitteldeutschlands in waldfreien oder bewaldeten Landschaften?. In: Schwarz K, editor. Strena Praehistorica. Festgabe zum 60. Geburtstag von Martin Jahn. Halle (Saale): Max Niemeyer; 1948. pp. 1–28.

[211] Schlüter O. Die Siedlungsräume Mitteleuropas in frühgeschichtlicher Zeit, 1: Einführung in die Methodik der Altlandschaftsforschung. Forschungen zur deutschen Landeskunde 63. Hamburg: Paul List; 1952.

[212] Schlüter O. Die Siedlungsräume Mitteleuropas in frühgeschichtlicher Zeit, 2: Erklärung und Begründung der Darstellung. Das südliche und nordwestliche Mitteleuropa. Forschungen zur deutschen Landeskunde 74. Hamburg: Paul List; 1953.

[213] Schlüter O, August O. Atlas des Saale- und mittleren Elbegebietes. Zweite, völlig neubearbeitete Auflage des Werkes Mitteldeutscher Heimatatlas. Leipzig: Verlag Enzyklopädie; 1959.

[214] Hiller A, Litt T, Eissmann L. Zur Entwicklung der jungquartären Tieflandstäler im Saale-Elbe-Raum unter besonderer Berücksichtigung von 14C-Daten. E & G Quaternary Science Journal. 1991; 41:26–46.

[215] Tinapp C. Entdeckungen am Grund des Elstermühlgrabens. Neue Ufer Leipzig. 2020; 12:40–3.

[216] Mania D. Zur spät- und nacheiszeitlichen Landschaftsgeschichte des mittleren Elb-Saalegebietes. Hallesches Jahrbuch für Mitteldeutsche Erdgeschichte. 1972; 11:7–36.

[217] Küster H. Sieben Phasen der Nutzung mitteleuropäischer Wälder. Alt Thuring. 1996; 30:55–70.

[218] Andraschko FM. Überlegungen zum Holzbedarf ur- und frühgeschichtlicher Siedlungen. Alt Thuring. 1996; 30:81–100.

[219] Kretschmer S, Viol P. Archäologische Voruntersuchungen im Abbaufeld Peres: Ein neues Kapitel der Braunkohlentagebauarchäologie im Südraum Leipzig. Ausgrabungen in Sachsen. 2012; 3:149–154.

[220] Kretschmer S, Viol P, Stäuble H. Ausgrabung eines linienbandkeramischen Fundplatzes bei Droßdorf (Lkr. Leipzig) im Tagebaufeld Peres: Ein erster Überblick. Ausgrabungen in Sachsen. 2014; 4:43–53.

[221] Stäuble H, Campen I. 7000 Jahre Brunnenbau im Südraum von Leipzig. In: Beyer B, editor. Brunnen der Jungsteinzeit: internationales Symposium in Erkelenz, 27. bis 29. Oktober 1997. Materialien zur Bodendenkmalpflege im Rheinland 11. Bonn: Habelt; 1998. pp. 51–71.

[222] Lies H. Zur neolithischen Siedlungsintensität im Magdeburger Raum. Jahresschrift für mitteldeutsche Vorgeschichte. 1974; 58: 57–111.

[223] Jäger KD. Ur- und frühgeschichtliche Klimabeeinflussung durch Intensitätsunterschiede agrarischer Landnutzung?. In: Cziesla E, Kersting T, Pratsch S, editors. Den Bogen Spannen…Teil 2: Festschrift für Bernhard Gramsch zum 65. Geburtstag. Beiträge zur Ur- und Frühgeschichte Mitteleuropas 20. Weissbach: Beier und Beran; 1999. pp. 515–522.

[224] Jäger KD. Klimawandel und Besiedlungsgeschichte in Mitteleuropa während der Nacheiszeit. Sitzungsberichte der Leibniz-Sozietät der Wissenschaften zu Berlin. 2009; 100:81–131.

