# Supplementary figures and images for: Large-scale investigations of Neolithic settlement dynamics in Central Germany based on machine learning analysis: A case study from the Weiße Elster river catchment
Source: PLoS One. 2022 Apr 20;17(4):e0265835. doi: 10.1371/journal.pone.0265835 (PMC9020700; doi:10.1371/journal.pone.0265835)

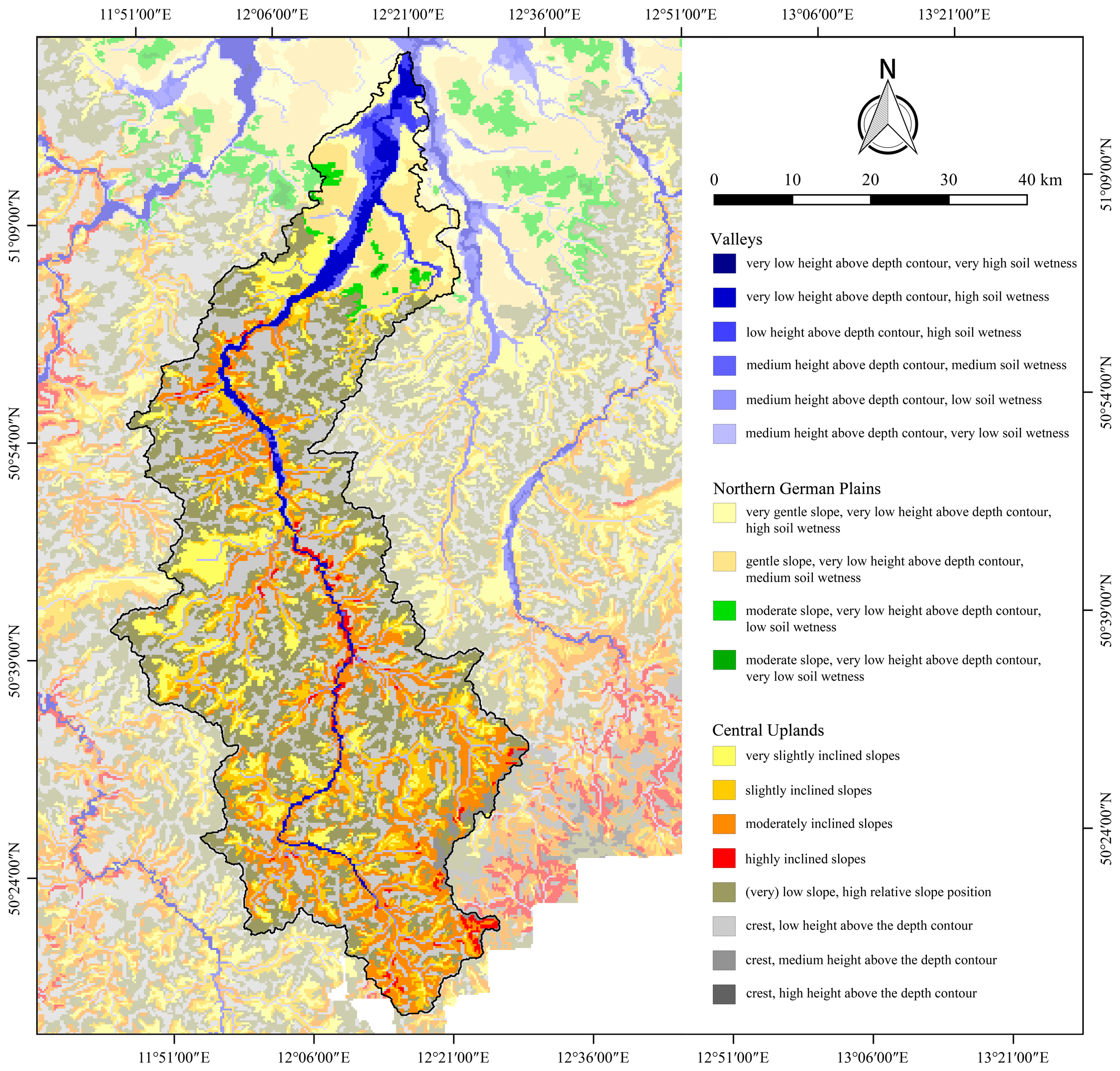

Supplement: S1 Fig — GMK1000RV2.0, (C) BGR, Hannover, 2006. Translated by Jan Miera. (TIF) [file pone.0265835.s010.tif]

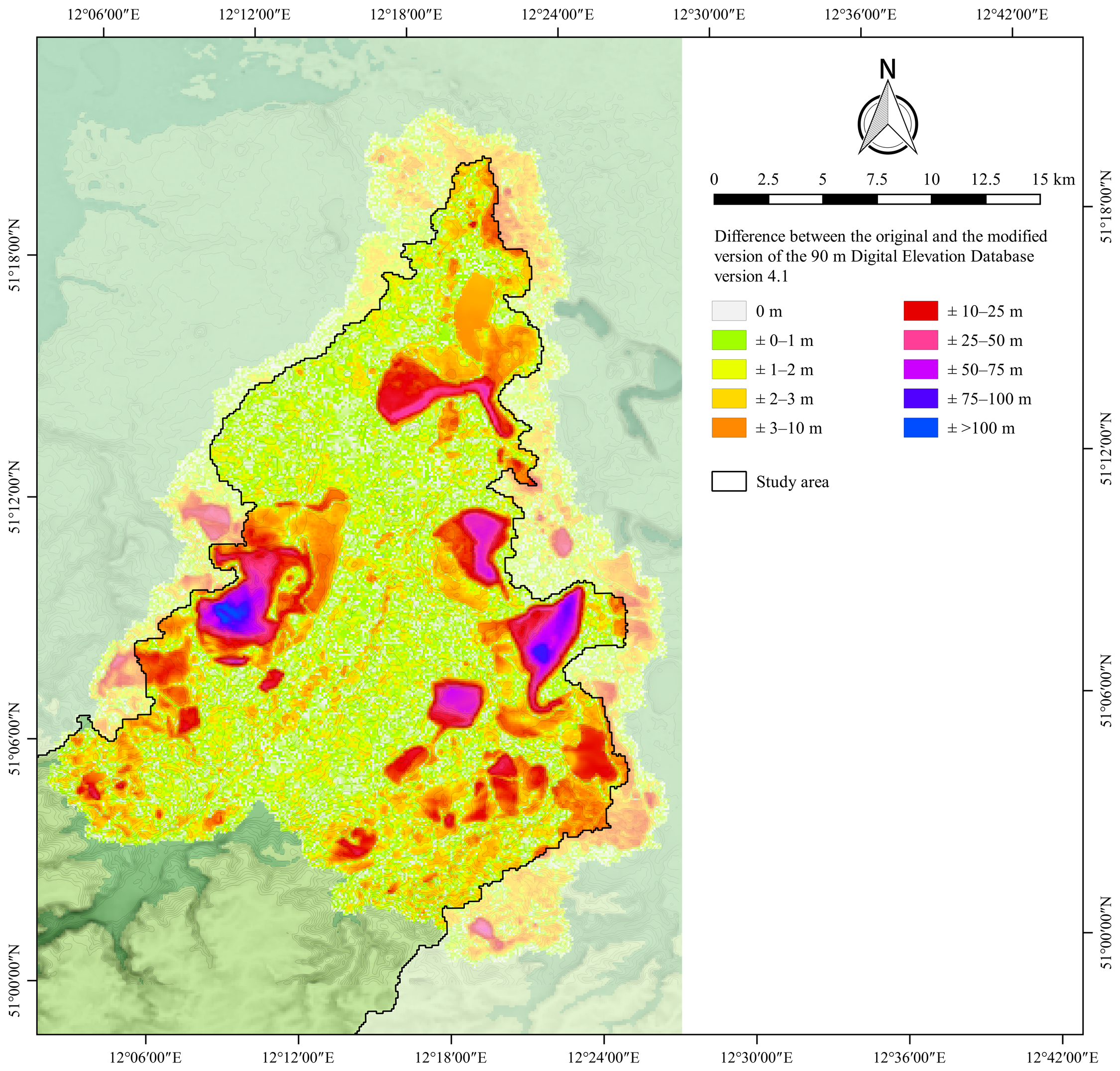

Supplement: S2 Fig — DEM data were provided by the SRTM Digital Elevation Database version 4.1 (Available from: http://srtm.csi.cgiar.org). (TIF) [file pone.0265835.s011.tif]

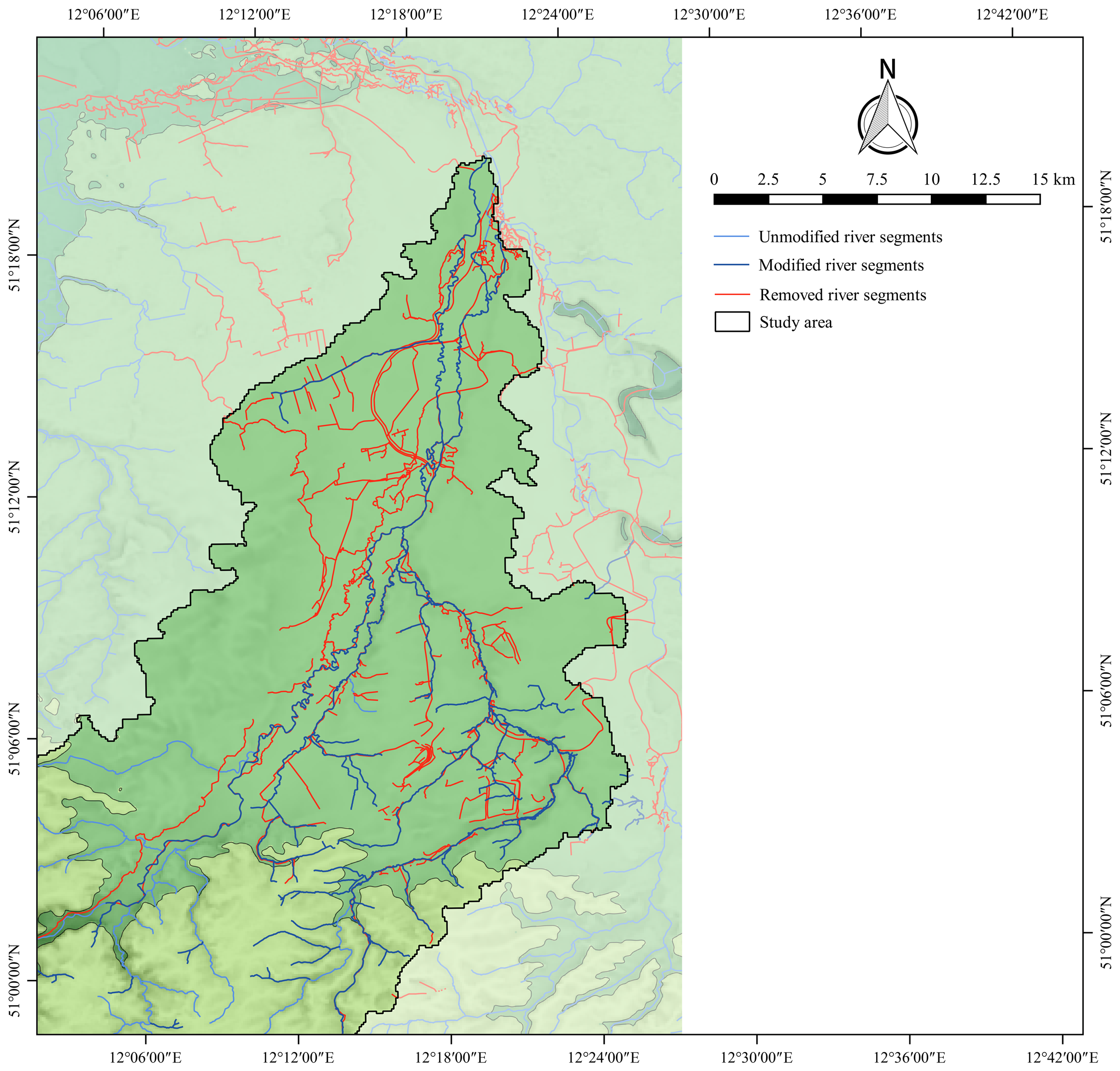

Supplement: S3 Fig — River data were provided by the Saxon State Office for Environment, Agriculture and Geology, the Saxony-Anhalt State Agency for Flood Protection and Water Management and the Thuringian State Office for Soil Management and Geoinformation. (TIF) [file pone.0265835.s012.tif]

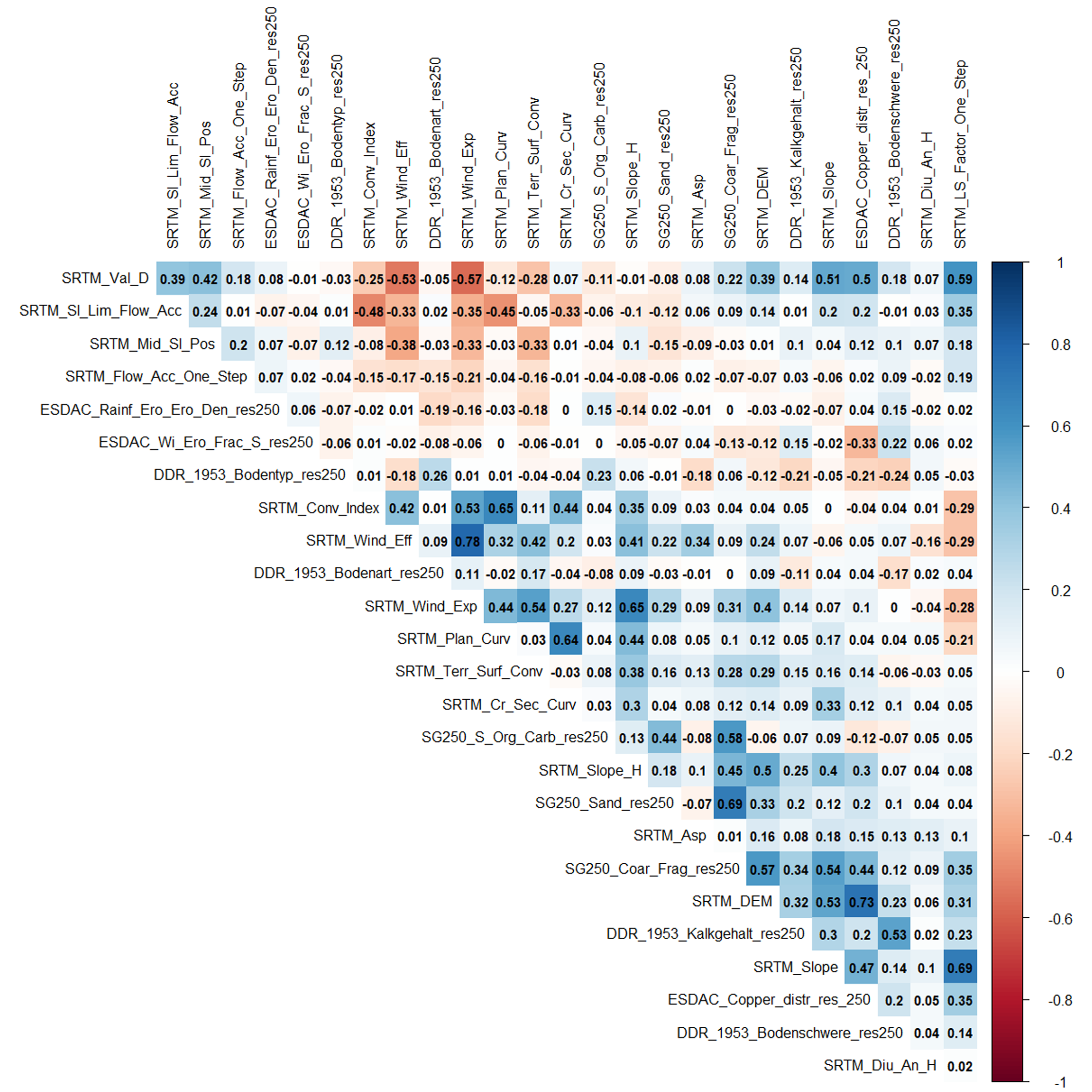

Supplement: S4 Fig — Here, we used R package corrplot from Wei T, Simko V. R package ’corrplot’: Visualization of a Correlation Matrix. (Version 0.92). 2021 [Cited 2022 February 1]. Available from: https://github.com/taiyun/corrplot. (TIF) [file pone.0265835.s013.tif]
