# Supplementary material for: Large-scale investigations of Neolithic settlement dynamics in Central Germany based on machine learning analysis: A case study from the Weiße Elster river catchment
Source: PLoS One. 2022 Apr 20;17(4):e0265835. doi: 10.1371/journal.pone.0265835 (PMC9020700; doi:10.1371/journal.pone.0265835)
Supplement: S1 References — (DOCX) [file pone.0265835.s017.docx]

# References cited only in the Supporting Information

Panagos P, Ballabio C, Borrelli P, Meusburger K, Klik A, Rousseva S, et al. Rainfall erosivity in Europe. Sci Total Environ. 2015; 511:801–814. https://doi.org/10.1016/j.scitotenv.2015.01.008

Borrelli P, Lugato E, Montanarella L, Panagos P. A New Assessment of Soil Loss Due to Wind Erosion in European Agricultural Soils Using a Quantitative Spatially Distributed Modelling Approach. Land Degrad Dev. 2017; 28:335–344. https://doi.org/10.1002/ldr.2588

Hengl T, Mendes de Jesus J, Heuvelink GBM, Ruiperez Gonzalez M, Kilibarda M, Blagotić A, et al. SoilGrids250m: Global gridded soil information based on machine learning. PLoS ONE. 2017; 12:e0169748. https://doi.org/10.1371/journal.pone.0169748

Poggio L, Sousa LM de, Batjes NH, Heuvelink GBM, Kempen B, Ribeiro E, et al. SoilGrids 2.0: producing soil information for the globe with quantified spatial uncertainty. SOIL. 2021; 7: 217–240. https://doi.org/10.5194/soil-7-217-2021
